# Supplementary material for: Five energy metabolism pathways show distinct regional distributions and lifespan trajectories in the human brain
Source: PLoS Biol. 2026 Jan 30;24(1):e3003619. doi: 10.1371/journal.pbio.3003619 (PMC12875592; doi:10.1371/journal.pbio.3003619)
Supplement: S1 Fig — Brain map depicts phosphogluconate dehydrogenase (PGD) gene expression according to the Schaefer-400 parcellation. Colorbar represents z-scored expression values. PGD expression was correlated (Spearman’s) with glycolysis and OXPHOS mean expression maps. Correlations were tested against a distribution of 10 000 correlations produced from the spatial permutation testing. The non-parametric p-value is indicated as pspin. Dots in the scatter plot represent 400 cortical regions in the Schaefer-400 parcellation. (PDF) [file pbio.3003619.s001.pdf]

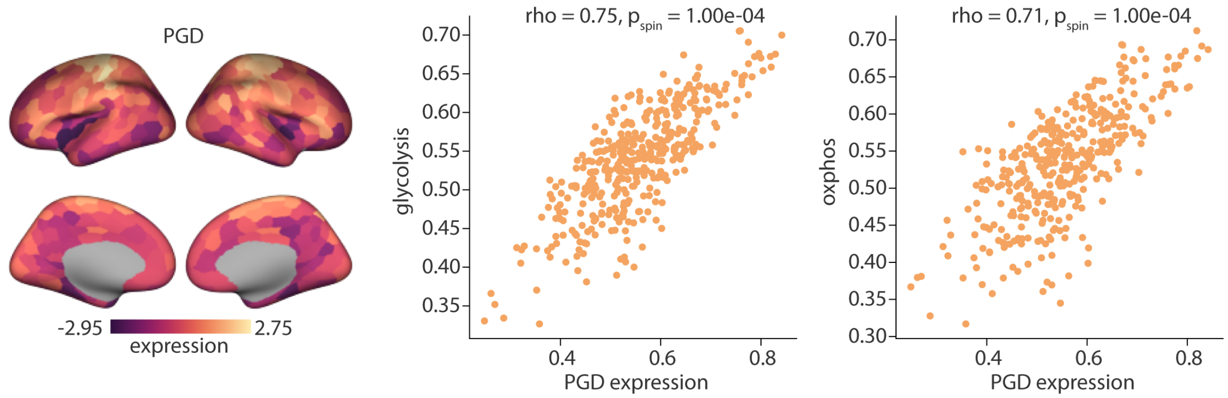

S1 Fig. **PGD gene expression correlates with glycolysis and OXPHOS maps.** Brain map depicts phosphogluconate dehydrogenase (*PGD*) gene expression according to the Schaefer-400 parcellation. Colorbar represents z-scored expression values. *PGD* expression was correlated (Spearman's) with glycolysis and OXPHOS mean expression maps. Correlations were tested against a distribution of 10 000 correlations produced from the spatial permutation testing. The non-parametric p-value is indicated as p_{spin}. Dots in the scatter plot represent 400 cortical regions in the Schaefer-400 parcellation.
