# Supplementary material for: Five energy metabolism pathways show distinct regional distributions and lifespan trajectories in the human brain
Source: PLoS Biol. 2026 Jan 30;24(1):e3003619. doi: 10.1371/journal.pbio.3003619 (PMC12875592; doi:10.1371/journal.pbio.3003619)
Supplement: S2 Fig — (a) Brain maps showing the first principal component (PC1) of pathway gene expression matrices. PC1 of the glycolysis and OXPHOS gene expression reflect a gradient from the motor and prefrontal cortices to the parietal association regions, and the visual cortex (glycolysis: varexplained=%45.46; OXPHOS: varexplained=%55.01). PC1 of the PPP gene expression shows a spatial pattern closely capturing the established global gene expression gradient, extending from the sensory cortices to the higher order association, and limbic areas [71] (varexplained=%32.30). The PC1 maps can be found in S1 Data. (b) Percent of variance explained by the first five principal components of pathway gene expression. (c) Spearman’s correlation between energy maps and the PC1 of expression of all genes in the AHBA. (d) Correlation between energy maps and average expression of all genes in the AHBA. Highlighted bars represent statistical significance when tested against a distribution of 10 000 spatial-autocorrelation preserving nulls (pspin<0.05). The correlation and pspin values can be found in S1 Data. ppp, pentose phosphate pathway; tca, tricarboxylic acid cycle; oxphos, oxidative phosphorylation; lactate, lactate metabolism and transport. (PDF) [file pbio.3003619.s002.pdf]

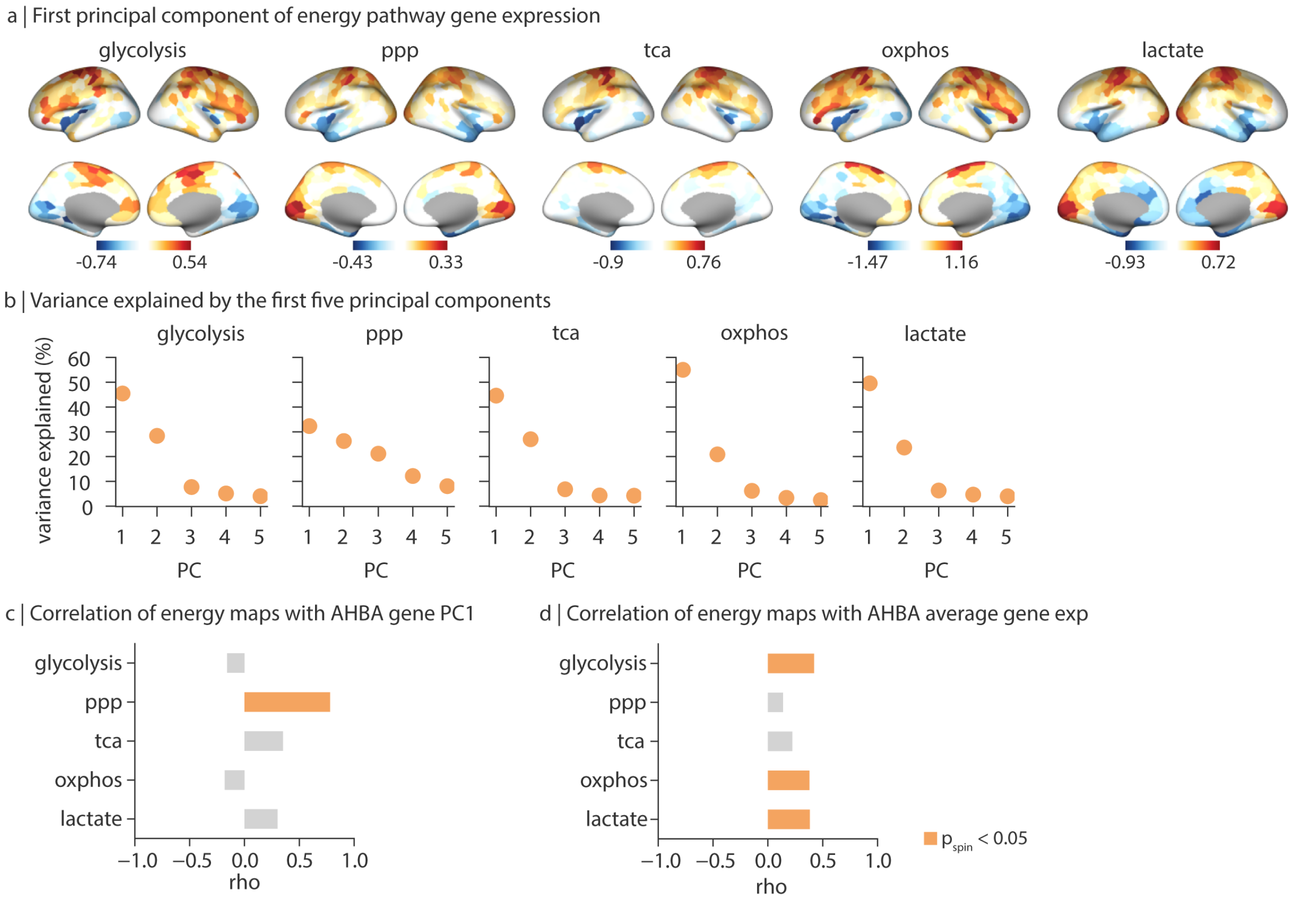

S2 Fig. **Principal component analysis of energy pathway gene expression.** (a) Brain maps showing the first principal component (PC1) of pathway gene expression matrices. PC1 of the glycolysis and OXPHOS gene expression reflect a gradient from the motor and prefrontal cortices to the parietal association regions, and the visual cortex (glycolysis: $var_{explained} = \%45.46$; OXPHOS: $var_{explained} = \%55.01$). PC1 of the PPP gene expression shows a spatial pattern closely capturing the established global gene expression gradient, extending from the sensory cortices to the higher order association, and limbic areas [1] ($var_{explained} = \%32.30$). The PC1 maps can be found in S1 Data. (b) Percent of variance explained by the first five principal components of pathway gene expression. (c) Spearman's correlation between energy maps and the PC1 of expression of all genes in the AHBA. (d) Correlation between energy maps and average expression of all genes in the AHBA. Highlighted bars represent statistical significance when tested against a distribution of 10 000 spatial-autocorrelation preserving nulls ($p_{\text{spin}} < 0.05$). The correlation and p_{spin} values can be found in S1 Data. ppp, pentose phosphate pathway; tca, tricarboxylic acid cycle; oxphos, oxidative phosphorylation; lactate, lactate metabolism and transport.

References

1. Burt JB, Demirtaş M, Eckner WJ, Navejar NM, Ji JL, Martin WJ, et al. Hierarchy of transcriptomic specialization across human cortex captured by structural neuroimaging topography. Nature Neuroscience. 2018 Sep;21(9):1251-9.
