# Supplementary material for: Five energy metabolism pathways show distinct regional distributions and lifespan trajectories in the human brain
Source: PLoS Biol. 2026 Jan 30;24(1):e3003619. doi: 10.1371/journal.pbio.3003619 (PMC12875592; doi:10.1371/journal.pbio.3003619)
Supplement: S3 Fig — Energy pathway gene expression matrices were retrieved for 14 subcortical regions in the Desikian-Killiany atlas [174]. Left: subcortical visualization of mean pathway gene expression. Ventricles are excluded due to the absence of gene expression data. Stable genes (ds≥0.1) were retained to produce pathway mean gene expression maps (see Methods). Colorbar represents expression values. Right: Barplot representation of the subcortical energy profiles (left hemisphere). Bars correspond to pathway mean gene expression, z-scored across all subcortical regions. Energy pathways consistently show higher expression in the thalamus and lower expression in the amygdala [55]. ppp, pentose phosphate pathway; tca, tricarboxylic acid cycle; oxphos, oxidative phosphorylation; lactate, lactate metabolism and transport. (PDF) [file pbio.3003619.s003.pdf]

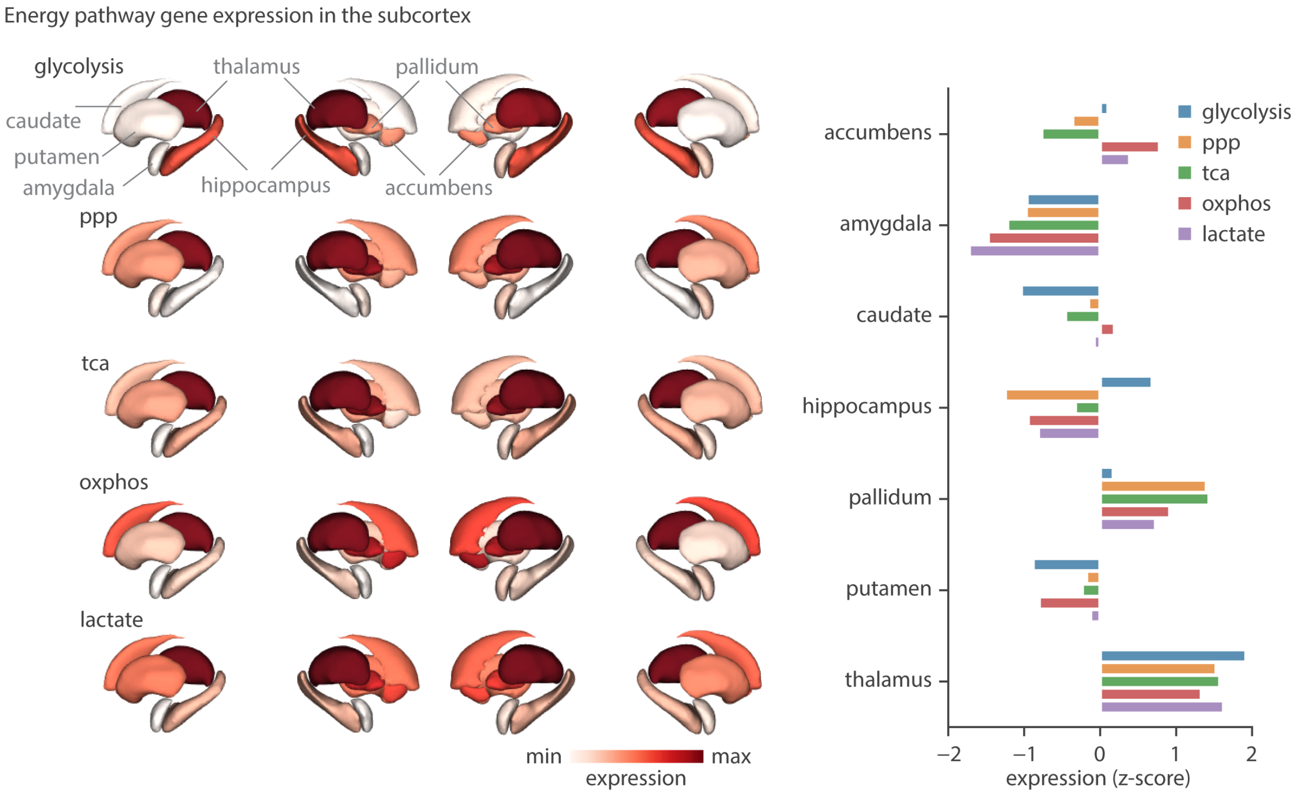

S3 Fig. **Subcortical energy pathway profiles.** Energy pathway gene expression matrices were retrieved for 14 subcortical regions in the Desikian-Killiany atlas [1]. Left: subcortical visualization of mean pathway gene expression. Ventricles are excluded due to the absence of gene expression data. Stable genes ($ds \geq 0.1$) were retained to produce pathway mean gene expression maps (see *Methods*). Colorbar represents expression values. Right: Barplot representation of the subcortical energy profiles (left hemisphere). Bars correspond to pathway mean gene expression, z-scored across all subcortical regions. Energy pathways consistently show higher expression in the thalamus and lower expression in the amygdala [2]. ppp, pentose phosphate pathway; tca, tricarboxylic acid cycle; oxphos, oxidative phosphorylation; lactate, lactate metabolism and transport.

References

1. Desikan RS, Ségonne F, Fischl B, Quinn BT, Dickerson BC, Blacker D, et al. An automated labeling system for subdividing the human cerebral cortex on MRI scans into gyral based regions of interest. NeuroImage. 2006 Jul;31(3):968-80.

2. Kleinridders A, Ferris HA, Reyzer ML, Rath M, Soto M, Manier ML, et al. Regional differences in brain glucose metabolism determined by imaging mass spectrometry. Molecular Metabolism. 2018 Jun;12:113-21.
