# Supplementary material for: Five energy metabolism pathways show distinct regional distributions and lifespan trajectories in the human brain
Source: PLoS Biol. 2026 Jan 30;24(1):e3003619. doi: 10.1371/journal.pbio.3003619 (PMC12875592; doi:10.1371/journal.pbio.3003619)
Supplement: S4 Fig — Maps were z-scored across the 400 cortical regions and the average expression of parcels falling into each functional network was calculated for each energy map, according to the Yeo-Kiernen intrinsic functional network parcellation [58]. Highlighted bars indicate statistical significance when tested against 10 000 spatial-autocorrelation preserving nulls (pspin<0.05). Brain plots visualize parcels making up each functional network. Glycolysis, TCA, OXPHOS and lactate maps show significantly greater values in the somato-motor cortex (glycolysis: pspin=0.049; TCA: pspin=0.0005; OXPHOS: pspin=0.03; lactate: pspin=0.02). Glycolysis and OXPHOS have significantly lower expressions in the visual cortex (glycolysis: pspin=0.003; OXPHOS: pspin=0.004). The PPP map on the other hand shows greater expression in the visual cortex, although not significant when tested against spatial permutations (pspin=0.09) and significantly lower expression in the limbic network (pspin=0.006). Data underlying this figure can be found in S1 Data. ppp, pentose phosphate pathway; tca, tricarboxylic acid cycle; oxphos, oxidative phosphorylation; lactate, lactate metabolism and transport. (PDF) [file pbio.3003619.s004.pdf]

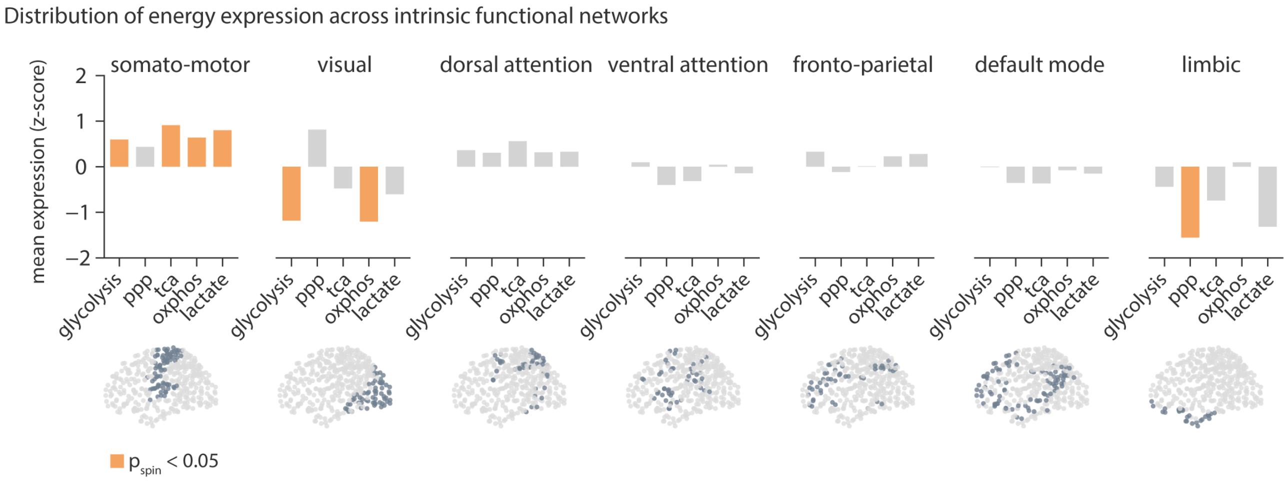

S4 Fig. **Distribution of energy pathway gene expression across intrinsic functional networks.** Maps were z-scored across the 400 cortical regions and the average expression of parcels falling into each functional network was calculated for each energy map, according to the Yeo-Kiernen intrinsic functional network parcellation [1]. Highlighted bars indicate statistical significance when tested against 10 000 spatial-autocorrelation preserving nulls ($p_{\text{spin}} < 0.05$). Brain plots visualize parcels making up each functional network. Glycolysis, TCA, OXPHOS and lactate maps show significantly greater values in the somato-motor cortex (glycolysis: $p_{\text{spin}} = 0.049$; TCA: $p_{\text{spin}} = 0.0005$; OXPHOS: $p_{\text{spin}} = 0.03$; lactate: $p_{\text{spin}} = 0.02$). Glycolysis and OXPHOS have significantly lower expressions in the visual cortex (glycolysis: $p_{\text{spin}} = 0.003$; OXPHOS: $p_{\text{spin}} = 0.004$). The PPP map on the other hand shows greater expression in the visual cortex, although not significant when tested against spatial permutations ($p_{\text{spin}} = 0.09$) and significantly lower expression in the limbic network ($p_{\text{spin}} = 0.006$). Data underlying this figure can be found in S1 Data. ppp, pentose phosphate pathway; tca, tricarboxylic acid cycle; oxphos, oxidative phosphorylation; lactate, lactate metabolism and transport.

References

1. Yeo BT, Krienen FM, Sepulcre J, Sabuncu MR, Lashkari D, Hollinshead M, et al. The organization of the human cerebral cortex estimated by intrinsic functional connectivity. Journal of Neurophysiology. 2011 Sep;106(3):1125-65.
