# Supplementary material for: Five energy metabolism pathways show distinct regional distributions and lifespan trajectories in the human brain
Source: PLoS Biol. 2026 Jan 30;24(1):e3003619. doi: 10.1371/journal.pbio.3003619 (PMC12875592; doi:10.1371/journal.pbio.3003619)
Supplement: S5 Fig — Mitochondrial phenotype maps were obtained from [75] and parcellated into Schaefer-400. The y-axis represents mitochondrial phenotype maps and the x-axis represents gene expression-based maps. Orange scatter plots indicate statistically significant correlations (Spearman’s) tested against 10 000 spatial-autocorrelation preserving nulls (pspin<0.05). CI, mitochondrial complex 1 activity; CII, mitochondrial complex 2 activity; CIV, mitochondrial complex 4 activity; MitoD, mitochondrial density; TRC, tissue respiratory capacity; MRC, mitochondrial respiratory capacity; tca, tricarboxylic acid cycle; oxphos, oxidative phosphorylation. (PDF) [file pbio.3003619.s005.pdf]

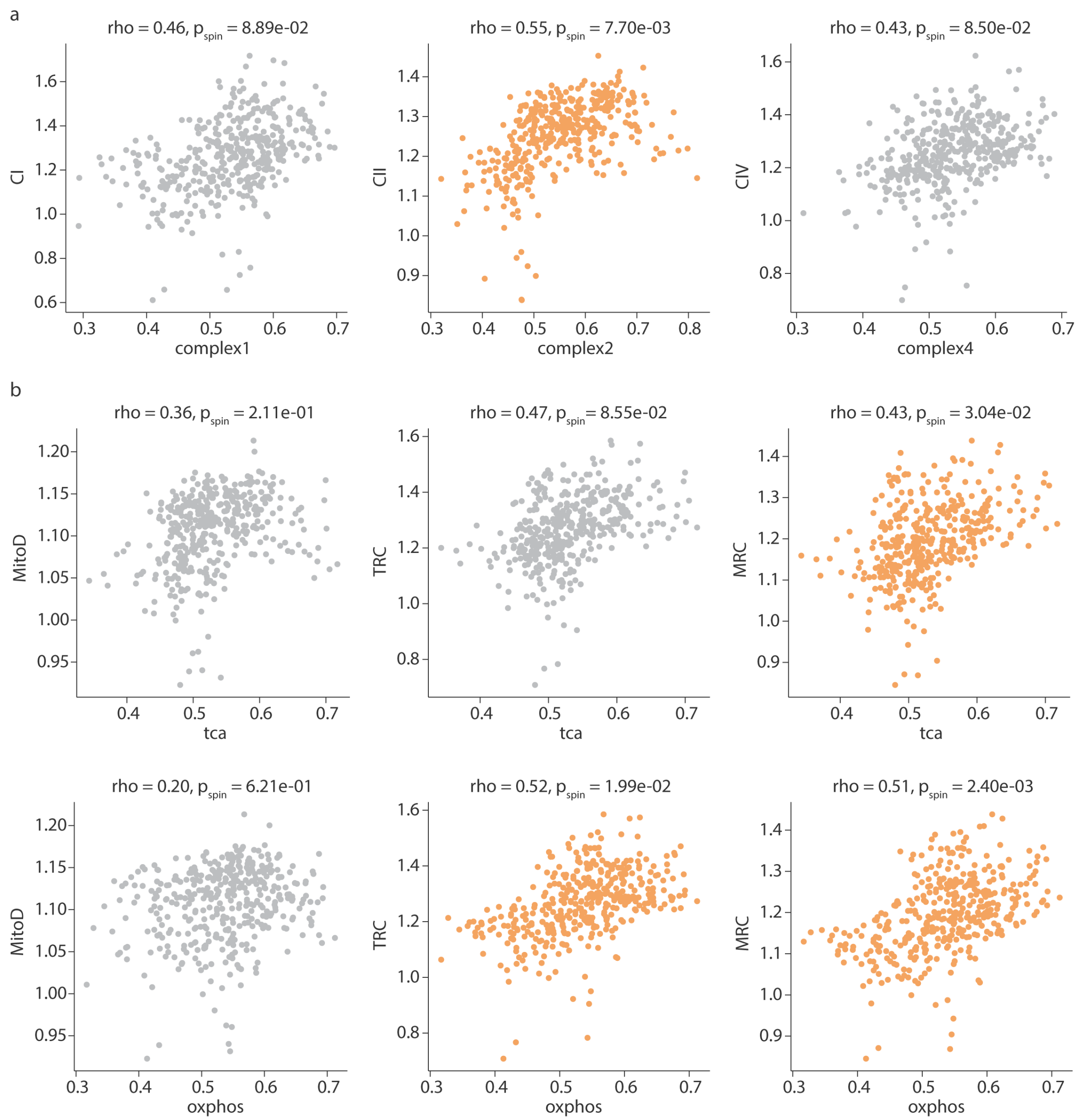

S5 Fig. **Correlation between energy gene expression and *ex-vivo* mitochondrial phenotype maps.** Mitochondrial phenotype maps were obtained from Mosharov et al. [1] and parcellated into Schaefer-400. The y-axis represents mitochondrial phenotype maps and the x-axis represents gene expression-based maps. Orange scatter plots indicate statistically significant correlations (Spearman's) tested against 10 000 spatial-autocorrelation preserving nulls ($p_{\text{spin}} < 0.05$). CI, mitochondrial complex 1 activity; CII, mitochondrial complex 2 activity; CIV, mitochondrial complex 4 activity; MitoD, mitochondrial density; TRC, tissue respiratory capacity; MRC, mitochondrial respiratory capacity; tca, tricarboxylic acid cycle; oxphos, oxidative phosphorylation.

References

1. Mosharov EV, Rosenberg AM, Monzel AS, Osto CA, Stiles L, Rosoklija GB, et al. A human brain map of mitochondrial respiratory capacity and diversity. Nature. 2025 Mar.
