# Supplementary material for: Five energy metabolism pathways show distinct regional distributions and lifespan trajectories in the human brain
Source: PLoS Biol. 2026 Jan 30;24(1):e3003619. doi: 10.1371/journal.pbio.3003619 (PMC12875592; doi:10.1371/journal.pbio.3003619)
Supplement: S6 Fig — For each individual sub-type, gene markers were obtained from [93] and [184]. For the list of gene markers see S3 Table. (Left) Heatmap showing the strength of correlation between energy and cell-type maps. Colorbar indicates Spearman’s correlation values. Asterisks indicate statistical significance when tested against a distribution of 10 000 spatial-autocorrelation preserving nulls after FDR-correction using the Benjamini-Hochberg method for multiple comparisons. (Right) Cortical distribution of neuronal subtype gene expression. Colorbar represents expression values across the 400 Schaefer regions. Cell types: pvalb, parvalbumin; sst, somatostatin; calb, calbindin; vip, vasoactive intestinal peptide; exc, excitatory. (PDF) [file pbio.3003619.s006.pdf]

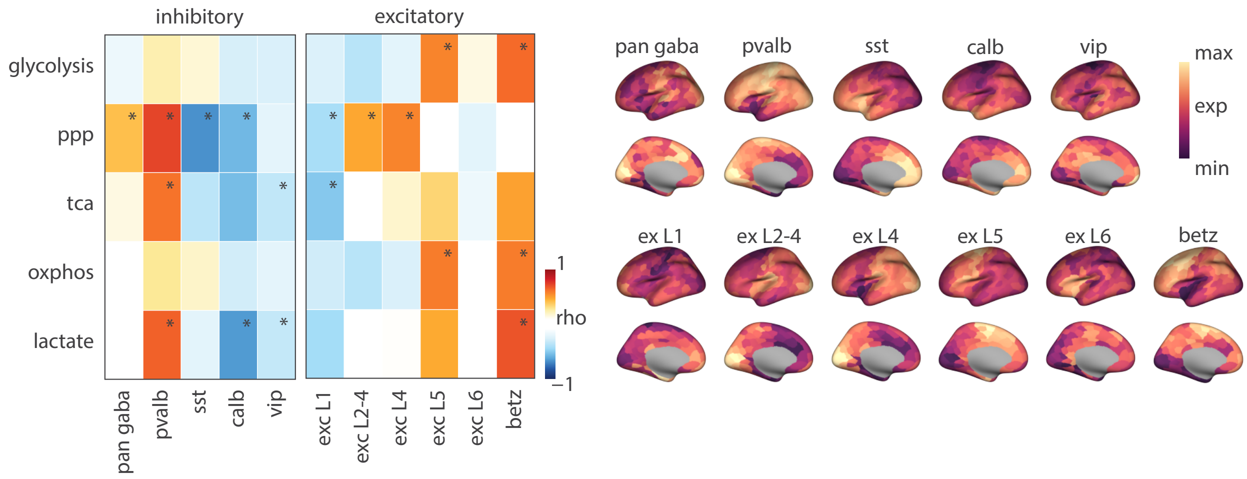

S6 Fig. **Spatial alignment between energy maps and individual inhibitory and excitatory subtypes.** For each individual subtype, gene markers were obtained from Kang et al. [1] and Hodge et al. [2]. For the list of gene markers see S3 Table. (Left) Heatmap showing the strength of correlation between energy and cell-type maps. Colorbar indicates Spearman's correlation values. Asterisks indicate statistical significance when tested against a distribution of 10 000 spatial-autocorrelation preserving nulls after FDR-correction using the Benjamini-Hochberg method for multiple comparisons. (Right) Cortical distribution of neuronal subtype gene expression. Colorbar represents expression values across the 400 Schaefer regions. CMR_{glc}, cerebral metabolic rate of glucose; CMR_{O2}, cerebral metabolic rate of oxygen; gi, glycolytic index; cbf, cerebral blood flow. Cell types: pvab, parvalbumin; sst, somatostatin; calb, calbindin; vip, vasoactive intestinal peptide; exc, excitatory

.

References

1. Kang HJ, Kawasawa YI, Cheng F, Zhu Y, Xu X, Li M, et al. Spatio-temporal transcriptome of the human brain. Nature. 2011 Oct;478(7370):483-9.
2. Hodge RD, Bakken TE, Miller JA, Smith KA, Barkan ER, Graybuck LT, et al. Conserved cell types with divergent features in human versus mouse cortex. Nature. 2019 Sep;573(7772):61-8.
