# Supplementary material for: Five energy metabolism pathways show distinct regional distributions and lifespan trajectories in the human brain
Source: PLoS Biol. 2026 Jan 30;24(1):e3003619. doi: 10.1371/journal.pbio.3003619 (PMC12875592; doi:10.1371/journal.pbio.3003619)
Supplement: S7 Fig — Expression trajectory of neurodevelopmental processes were produced using previously curated gene sets [88,93]. Mean expression of marker genes for each developmental process was calculated for each sample. Samples were then grouped into age categories. Line plot represents median values across all samples in each age group. Analysis only included cortical regions. The y-axis represents upper quartile normalized log2(RPKM) values (see Methods). Dots represent individual samples in each age group. For details of ages included in each group see S4 Table. (PDF) [file pbio.3003619.s007.pdf]

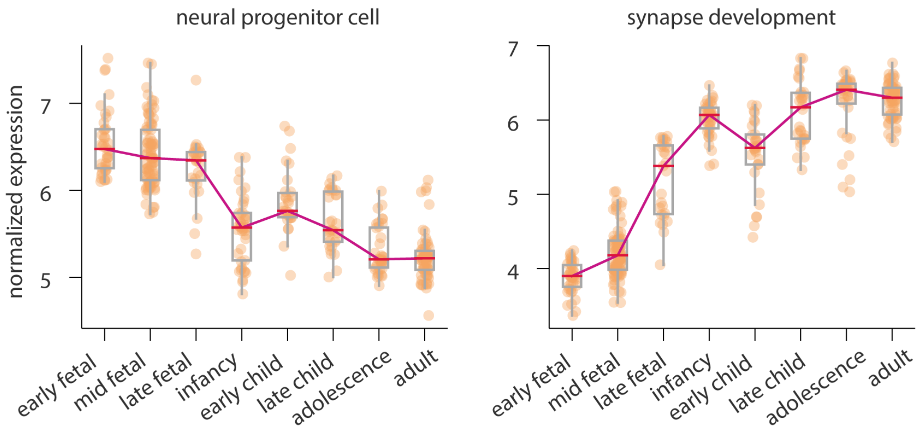

S7 Fig. **Lifespan trajectory of genes related to neurodevelopmental processes.** Expression trajectory of neurodevelopmental processes were produced using previously curated gene sets [1, 2]. Mean expression of marker genes for each developmental process was calculated for each sample. Samples were then grouped into age categories. Line plot represents median values across all samples in each age group. Analysis only included cortical regions. The y-axis represents upper quartile normalized $\log_2$(RPKM) values (see *Methods*). Dots represent individual samples in each age group. For details of ages included in each group see S4 Table.

References

1. Kang HJ, Kawasawa YI, Cheng F, Zhu Y, Xu X, Li M, et al. Spatio-temporal transcriptome of the human brain. Nature. 2011 Oct;478(7370):483-9.

2. Li M, Santpere G, Imamura Kawasawa Y, Evgrafov OV, Gulden FO, Pochareddy S, et al. Integrative functional genomic analysis of human brain development and neuropsychiatric risks. Science. 2018 Dec;362(6420).
