# Supplementary material for: Five energy metabolism pathways show distinct regional distributions and lifespan trajectories in the human brain
Source: PLoS Biol. 2026 Jan 30;24(1):e3003619. doi: 10.1371/journal.pbio.3003619 (PMC12875592; doi:10.1371/journal.pbio.3003619)
Supplement: S8 Fig — Lifespan analysis was done using the non-parametric locally estimated scatterplot smoothing (LOESS) method. For each energy pathway, mean expression was calculated across all genes for each sample. Samples were grouped into 11 cortical regions available in the dataset. The x-axis represent log10 transformed age in post conception days. Dots represent individual cortical samples at each age colored by region. The y-axis shows upper quartile normalized log2(RPKM) values. ppp, pentose phosphate pathway; tca, tricarboxylic acid cycle; oxphos, oxidative phosphorylation; lactate, lactate metabolism and transport. (PDF) [file pbio.3003619.s008.pdf]

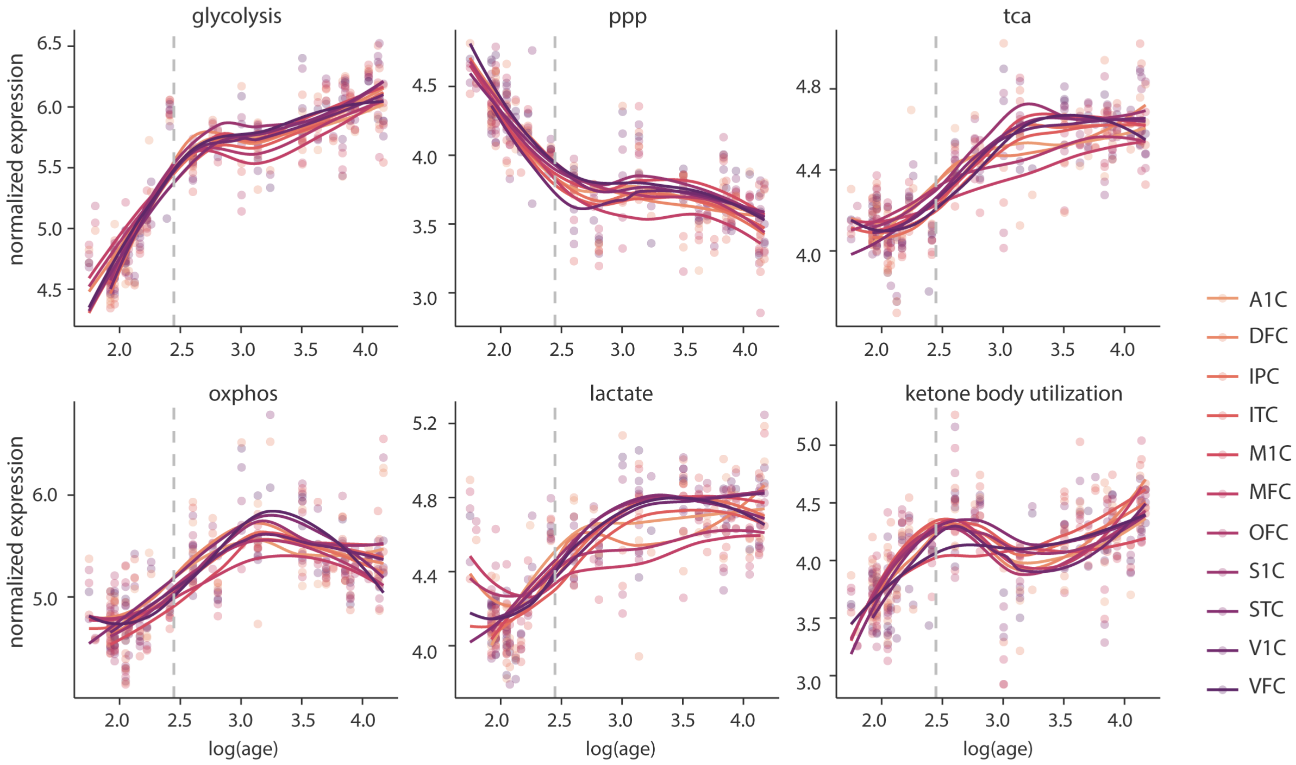

S8 Fig. **Region-wise developmental trajectory of energy maps.** Lifespan analysis was done using the non-parametric locally estimated scatterplot smoothing (LOESS) method. For each energy pathway, mean expression was calculated across all genes for each sample. Samples were grouped into 11 cortical regions available in the dataset. The x-axis represent $\log_{10}$ transformed age in post conception days. Dots represent individual cortical samples at each age colored by region. The y-axis shows upper quartile normalized $\log_2(\mathrm{RPKM})$ values. ppp, pentose phosphate pathway; tca, tricarboxylic acid cycle; oxphos, oxidative phosphorylation; lactate, lactate metabolism and transport.
