# Supplementary material for: Five energy metabolism pathways show distinct regional distributions and lifespan trajectories in the human brain
Source: PLoS Biol. 2026 Jan 30;24(1):e3003619. doi: 10.1371/journal.pbio.3003619 (PMC12875592; doi:10.1371/journal.pbio.3003619)
Supplement: S9 Fig — To assess the robustness of the results, energy maps were reproduced using (1) only the left hemisphere microarray data, and (2) a coarser parcellation (Schaefer-100). (a) Maps produced using only left hemisphere data and their correlation with the left hemisphere of maps in the main analysis (produced by mirroring across hemispheres). Scatter plot represents 200 regions in the left hemisphere parcellated according to Schaefer-400 parcellation. (b) Energy maps according to Schaefer-100 parcellation. Left: Heatmap depicts the pairwise correlation among all genes included in the energy sets across 100 regions in the Schaefer-100 parcellation. Middle: Spearman’s correlation between mean expression energy maps. Right: correlation of energy maps with the first principal component of all genes in AHBA (gene PC1). Brain map color bars represent z-scored expression across all regions in each parcellation. Highlighted bars show statistical significance when tested against 1 000 spatial-autocorrelation preserving nulls. Data underlying this figure can be found in S1 Data. lh, left hemisphere; ppp, pentose phosphate pathway; tca, tricarboxylic acid cycle; oxphos, oxidative phosphorylation; lactate, lactate metabolism and transport. (PDF) [file pbio.3003619.s009.pdf]

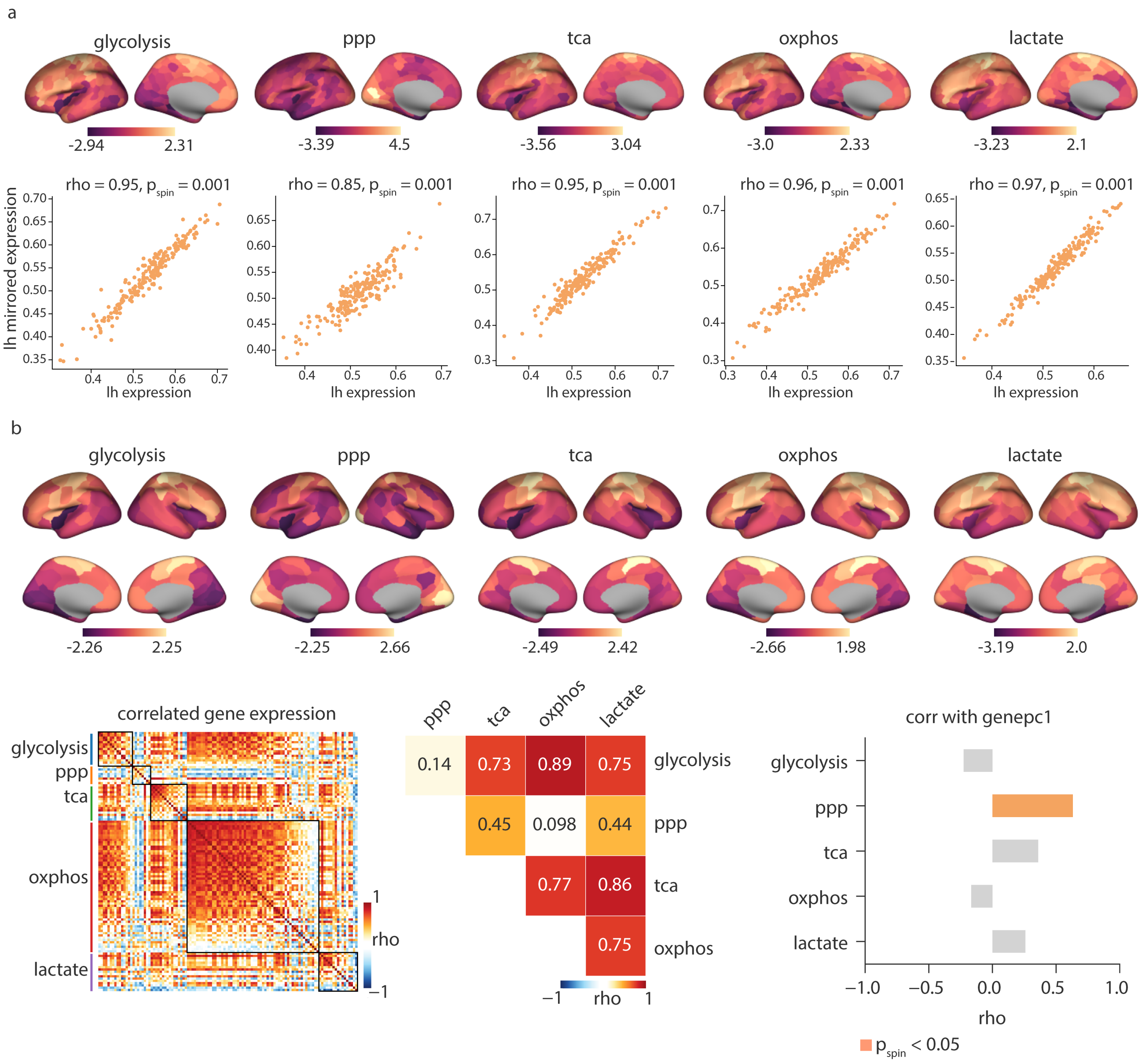

S9 Fig. **Sensitivity analysis.** To assess the robustness of the results, energy maps were reproduced using (1) only the left hemisphere microarray data, and (2) a coarser parcellation (Schaefer-100). (a) Maps produced using only left hemisphere data and their correlation with the left hemisphere of maps in the main analysis (produced by mirroring across hemispheres). Scatter plot represents 200 regions in the left hemisphere parcellated according to Schaefer-400 parcellation. (b) Energy maps according to Schaefer-100 parcellation. Left: Heatmap depicts the pairwise correlation among all genes included in the energy sets across 100 regions in the Schaefer-100 parcellation. Middle: Spearman's correlation between mean expression energy maps. Right: correlation of energy maps with the first principal component of all genes in AHBA (gene PC1). Brain map color bars represent z-scored expression across all regions in each parcellation. Highlighted bars show statistical significance when tested against 1 000 spatial-autocorrelation preserving nulls. Data underlying this figure can be found in S1 Data. lh, left hemisphere; ppp, pentose phosphate pathway; tca, tricarboxylic acid cycle; oxphos, oxidative phosphorylation; lactate, lactate metabolism and transport.
