# Supplementary material for: Five energy metabolism pathways show distinct regional distributions and lifespan trajectories in the human brain
Source: PLoS Biol. 2026 Jan 30;24(1):e3003619. doi: 10.1371/journal.pbio.3003619 (PMC12875592; doi:10.1371/journal.pbio.3003619)
Supplement: S10 Fig — Energy maps were reproduced using all available energy genes in the AHBA regardless of their differential stability threshold. (a) Spearman’s correlation between ds≥0.1 maps and maps with no differential stability threshold. Correlations were tested against a distribution of 10 000 nulls produced from the spatial permutation testing. The non-parametric p-value is indicated as pspin. Dots represent 400 cortical regions in the Schaefer-400 parcellation. (b) Left: Heatmap depicts Spearman’s correlation between mean expression energy maps. Middle: correlation of energy maps with the first principal component of all genes in AHBA (gene pc1). Left: Alignment between the FC gradients and energy maps. (c) Top: enrichment of energy maps across the seven von Economo cytoacrhitectonics classes. Bottom: enrichment of energy maps across the Mesulam sensory-fugal axis of information processing. The y-axis represents mean gene expression of z-scored maps. Highlighted bars indicate statistical significance (pspin<0.05). Data for this figure is provided in S1 Data. ds, differential stability; ppp, pentose phosphate pathway; tca, tricarboxylic acid cycle; oxphos, oxidative phosphorylation; lactate, lactate metabolism and transport. (PDF) [file pbio.3003619.s010.pdf]

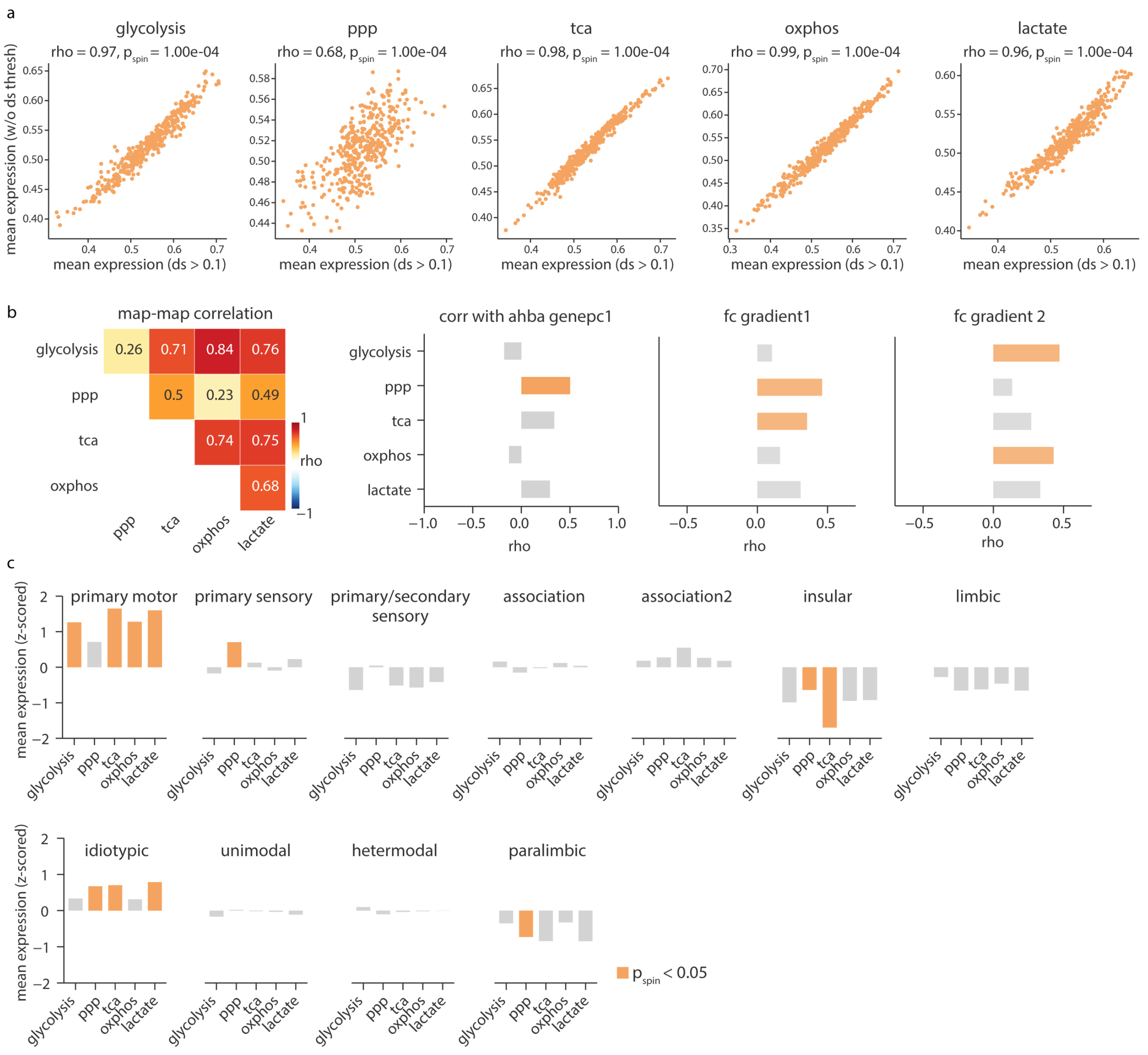

S10 Fig. **Energy maps without the differential stability threshold.** Energy maps were reproduced using all available energy genes in the AHBA regardless of their differential stability threshold. (a) Spearman's correlation between $ds \geq 0.1$ maps and maps with no differential stability threshold. Correlations were tested against a distribution of 10 000 nulls produced from the spatial permutation testing. The non-parametric p-value is indicated as p_{spin}. Dots represent 400 cortical regions in the Schaefer-400 parcellation. (b) Left: Heatmap depicts Spearman's correlation between mean expression energy maps. Middle: correlation of energy maps with the first principal component of all genes in AHBA (gene pc1). Left: Alignment between the FC gradients and energy maps. (c) Top: enrichment of energy maps across the seven von Economo cytoacrhitectonics classes. Bottom: enrichment of energy maps across the Mesulam sensory-fugal axis of information processing. The y-axis represents mean gene expression of z-scored maps. Highlighted bars indicate statistical significance ($p_{\text{spin}} < 0.05$). Data for this figure is provided in S1 Data. ds, differential stability; ppp, pentose phosphate pathway; tca, tricarboxylic acid cycle; oxphos, oxidative phosphorylation; lactate, lactate metabolism and transport.
