# Supplementary material for: Five energy metabolism pathways show distinct regional distributions and lifespan trajectories in the human brain
Source: PLoS Biol. 2026 Jan 30;24(1):e3003619. doi: 10.1371/journal.pbio.3003619 (PMC12875592; doi:10.1371/journal.pbio.3003619)
Supplement: S11 Fig — (a) Alignment between the mean gene expression maps and the first principal component of gene expression for each energy pathway. Dots in the scatterplot represent 400 cortical regions in the Schaefer-400 parcellation. (b) Spatial alignment among energy PC1 maps calculated using pairwise Spearman’s correlation. (c) Spearman’s correlation of PC1 energy maps and multi-scale cortical features. Asterisk represents significance after permutation testing using 10 000 spatial nulls and FDR correction using the Benjamini-Hochberg method for multiple comparison. Colorbar shows Spearman’s correlation values. CMRglc, cerebral metabolic rate of glucose; CMRO2, cerebral metabolic rate of oxygen; gi, glycolytic index; cbf, cerebral blood flow. Cell types: astro, astrocyte; neuron ex, excitatory neuron; neuron in, inhibitory neuron; endo, endothelial cell; micro, microglia; opc, oligodendrocyte precursor cells; oligo, oligodendrocyte; sc, structural connectivity; fc: functional connectivity. (PDF) [file pbio.3003619.s011.pdf]

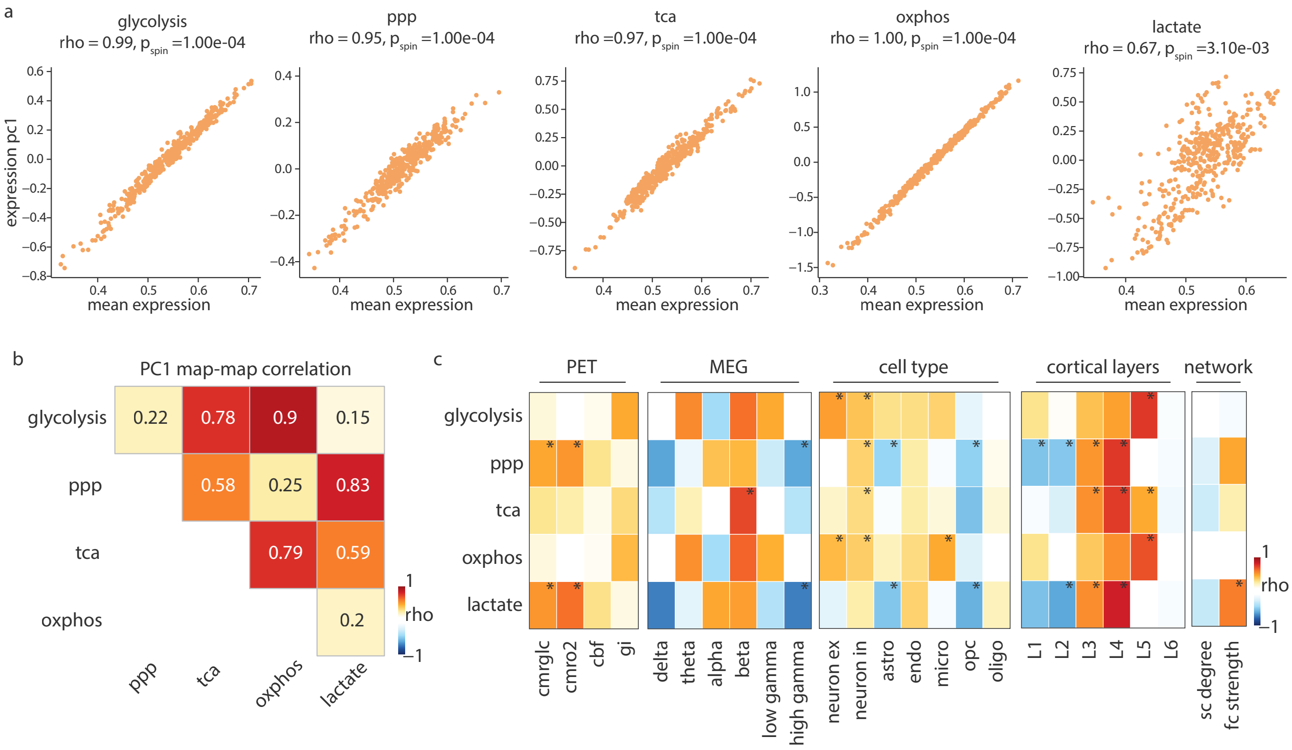

S11 Fig. **Energy maps produced from the first principal component of pathway gene expression.** (a) Alignment between the mean gene expression maps and the first principal component of gene expression for each energy pathway. Dots in the scatterplot represent 400 cortical regions in the Schaefer-400 parcellation. (b) Spatial alignment among energy PC1 maps calculated using pairwise Spearman's correlation. (c) Spearman's correlation of PC1 energy maps and multi-scale cortical features. Asterisk represents significance after permutation testing using 10 000 spatial nulls and FDR correction using the Benjamini-Hochberg method for multiple comparison. Colorbar shows Spearman's correlation values. CMR_{glc}, cerebral metabolic rate of glucose; CMR_{O2}, cerebral metabolic rate of oxygen; gi, glycolytic index; cbf, cerebral blood flow. Cell types: astro, astrocyte; neuron ex, excitatory neuron; neuron in, inhibitory neuron; endo, endothelial cell; micro, microglia; opc, oligodendrocyte precursor cells; oligo, oligodendrocyte; sc, structural connectivity; fc: functional connectivity.
