# Supplementary material for: Five energy metabolism pathways show distinct regional distributions and lifespan trajectories in the human brain
Source: PLoS Biol. 2026 Jan 30;24(1):e3003619. doi: 10.1371/journal.pbio.3003619 (PMC12875592; doi:10.1371/journal.pbio.3003619)
Supplement: S12 Fig — Gene expression matrices were retrieved for each subject using abagen package. Genes with ds≥0.1 were kept and pathway mean gene expression maps were produced as before (See methods. For each pathway, the heatmap depicts Spearman’s correlation between individual subjects. The x- and y-axis corresponds to the donor IDs. ppp, pentose phosphate pathway; tca, tricarboxylic acid cycle; oxphos, oxidative phosphorylation; lactate, lactate metabolism and transport. (PDF) [file pbio.3003619.s012.pdf]

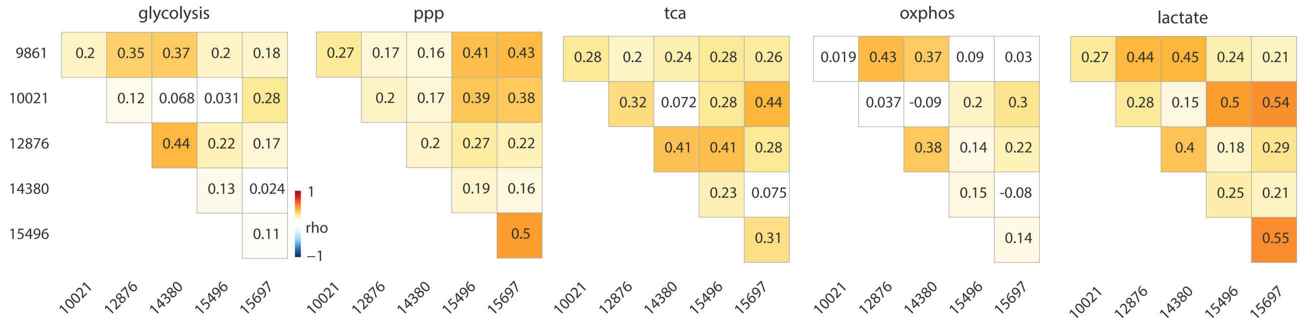

S12 Fig. **Correlation between subject level energy maps.** Gene expression matrices were retrieved for each subject using *abagen* package. Genes with $ds \geq 0.1$ were kept and pathway mean gene expression maps were produced as before (*See methods*. For each pathway, the heatmap depicts Spearman's correlation between individual subjects. The x- and y-axis corresponds to the donor IDs. ppp, pentose phosphate pathway; tca, tricarboxylic acid cycle; oxphos, oxidative phosphorylation; lactate, lactate metabolism and transport.
