# Supplementary material for: Five energy metabolism pathways show distinct regional distributions and lifespan trajectories in the human brain
Source: PLoS Biol. 2026 Jan 30;24(1):e3003619. doi: 10.1371/journal.pbio.3003619 (PMC12875592; doi:10.1371/journal.pbio.3003619)
Supplement: S13 Fig — Maps were z-scored across the 400 cortical regions and the average expression of parcels falling into each class was calculated for each energy pathway. The y-axis represents mean gene expression of z-scored maps. Highlighted bars indicate statistical significance when tested against 10 000 spatial-autocorrelation preserving nulls (pspin<0.05). Data for this figure can be found in S1 Data. ppp, pentose phosphate pathway; tca, tricarboxylic acid cycle; oxphos, oxidative phosphorylation; lactate, lactate metabolism and transport. (PDF) [file pbio.3003619.s013.pdf]

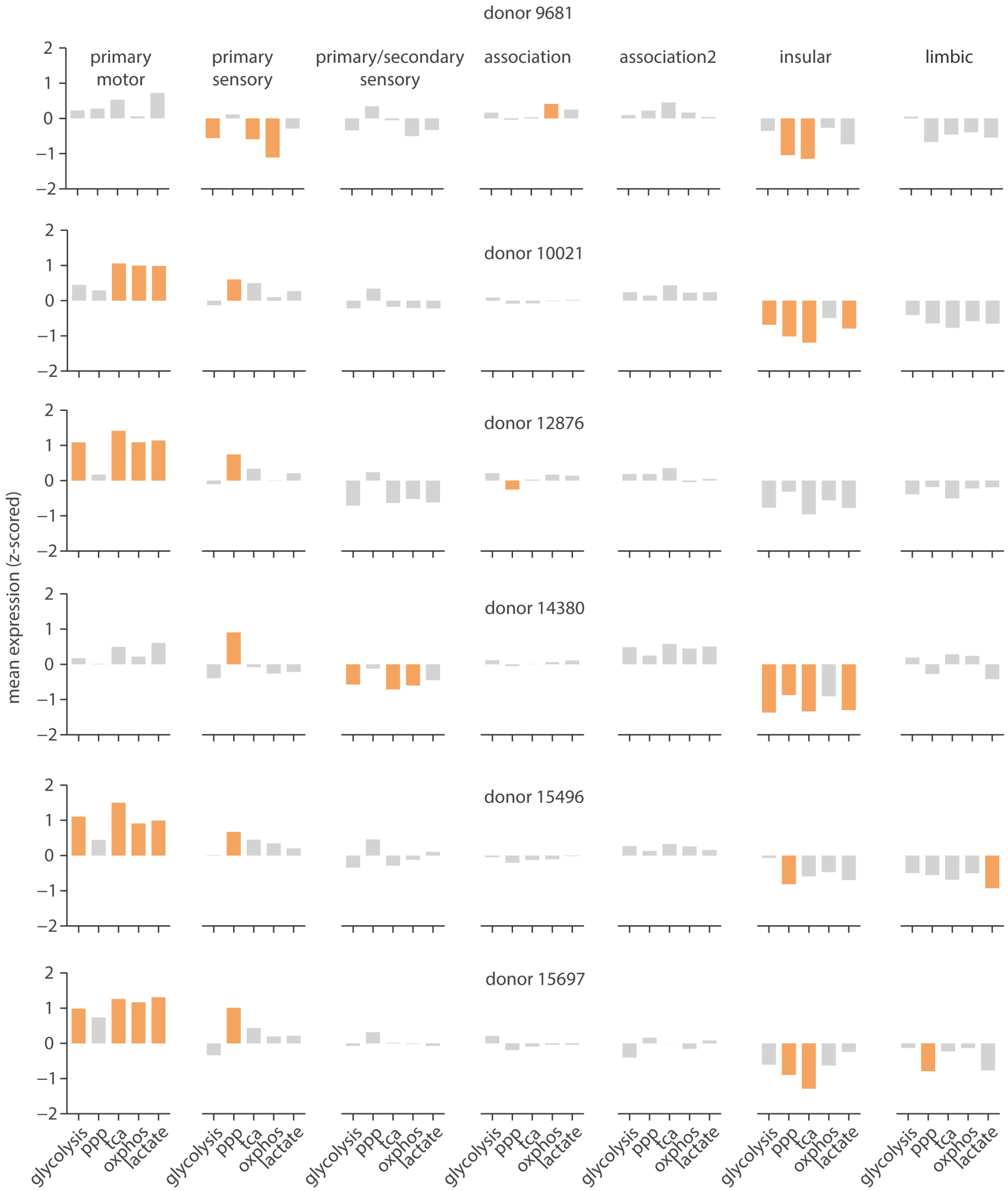

S13 Fig. **Distribution of subject-level energy gene expression across the von Economo classes.** Maps were z-scored across the 400 cortical regions and the average expression of parcels falling into each class was calculated for each energy pathway. The y-axis represents mean gene expression of z-scored maps. Highlighted bars indicate statistical significance when tested against 10 000 spatial-autocorrelation preserving nulls ($p_{\text{spin}} < 0.05$). Data for this figure can be found in S1 Data. ppp, pentose phosphate pathway; tca, tricarboxylic acid cycle; oxphos, oxidative phosphorylation; lactate, lactate metabolism and transport.
