# Supplementary material for: Five energy metabolism pathways show distinct regional distributions and lifespan trajectories in the human brain
Source: PLoS Biol. 2026 Jan 30;24(1):e3003619. doi: 10.1371/journal.pbio.3003619 (PMC12875592; doi:10.1371/journal.pbio.3003619)
Supplement: S14 Fig — Lifespan analysis was repeated using the non-parametric locally estimated scatterplot smoothing (LOESS) method. For each energy pathway, mean expression was calculated across all genes for each sample. The x-axis represent log10 transformed age in post conception days. Dots represent individual cortical samples at each age. The y-axis shows upper quartile normalized log2(RPKM) values. ppp, pentose phosphate pathway; tca, tricarboxylic acid cycle; oxphos, oxidative phosphorylation; lactate, lactate metabolism and transport. (PDF) [file pbio.3003619.s014.pdf]

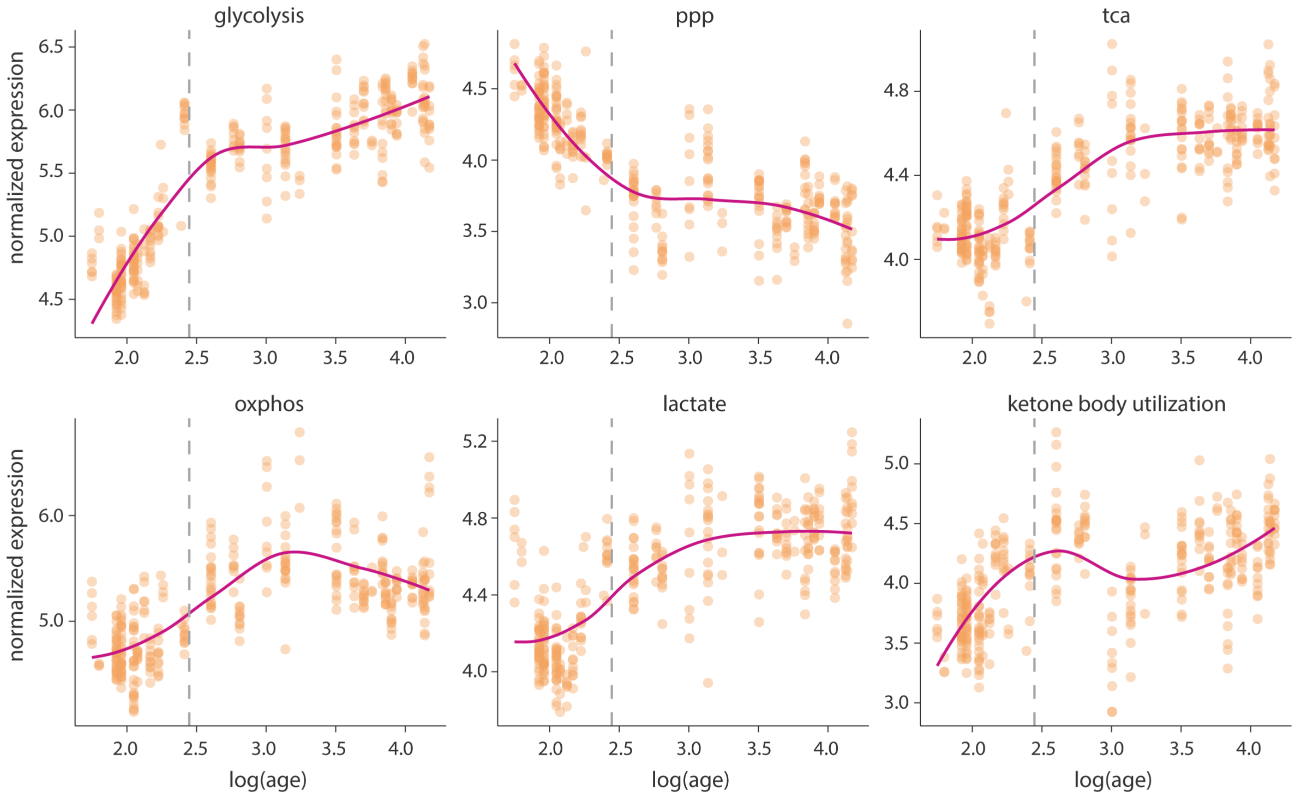

S14 Fig. **Lifespan trajectory of energy maps using LOESS.** Lifespan analysis was repeated using the non-parametric locally estimated scatterplot smoothing (LOESS) method. For each energy pathway, mean expression was calculated across all genes for each sample. The x-axis represent $\log_{10}$ transformed age in post conception days. Dots represent individual cortical samples at each age. The y-axis shows upper quartile normalized $\log_2(\text{RPKM})$ values. ppp, pentose phosphate pathway; tca, tricarboxylic acid cycle; oxphos, oxidative phosphorylation; lactate, lactate metabolism and transport.
