# Supplementary material for: Five energy metabolism pathways show distinct regional distributions and lifespan trajectories in the human brain
Source: PLoS Biol. 2026 Jan 30;24(1):e3003619. doi: 10.1371/journal.pbio.3003619 (PMC12875592; doi:10.1371/journal.pbio.3003619)
Supplement: S15 Fig — Lifespan analysis was repeated using the microarray data from the BrainSpan dataset [93]. For each energy pathway, mean expression was calculated across all genes for each sample. Samples were then grouped into eight age bins and the median expression across all samples falling into each age bin was calculated. Analysis only included cortical regions. The y-axis represents upper quartile normalized log2(signal intensity) (see Methods). Dots represent individual samples in each age group. Line plot depicts the trajectory of median gene expression. For details of ages included in each group see S8 Table. ppp, pentose phosphate pathway; tca, tricarboxylic acid cycle; oxphos, oxidative phosphorylation; lactate, lactate metabolism and transport. (PDF) [file pbio.3003619.s015.pdf]

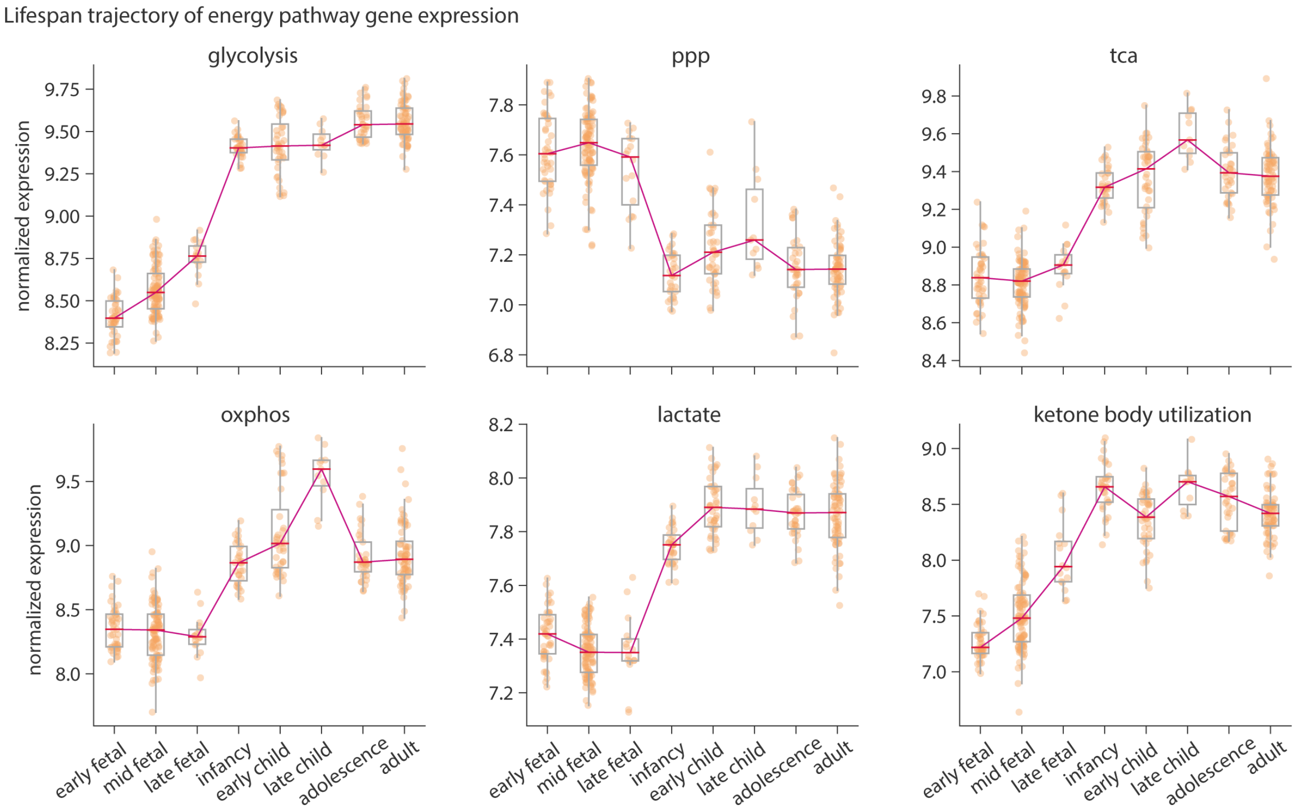

S15 Fig. **Microarray expression trajectory of energy maps across the lifespan.** Lifespan analysis was repeated using the microarray data from the BrainSpan dataset [1]. For each energy pathway, mean expression was calculated across all genes for each sample. Samples were then grouped into eight age bins and the median expression across all samples falling into each age bin was calculated. Analysis only included cortical regions. The y-axis represents upper quartile normalized $\log_2$(signal intensity) (see *Methods*). Dots represent individual samples in each age group. Line plot depicts the trajectory of median gene expression. For details of ages included in each group see S8 Table. ppp, pentose phosphate pathway; tca, tricarboxylic acid cycle; oxphos, oxidative phosphorylation; lactate, lactate metabolism and transport.

References

1. Kang HJ, Kawasawa YI, Cheng F, Zhu Y, Xu X, Li M, et al. Spatio-temporal transcriptome of the human brain. Nature. 2011 Oct;478(7370):483-9.
