# Supplementary material for: Five energy metabolism pathways show distinct regional distributions and lifespan trajectories in the human brain
Source: PLoS Biol. 2026 Jan 30;24(1):e3003619. doi: 10.1371/journal.pbio.3003619 (PMC12875592; doi:10.1371/journal.pbio.3003619)
Supplement: S16 Fig — Mean gene expression maps were produced as before (see Methods). Color bars represent mean gene expression, z-scored across the 400 cortical regions in the Schaefer-400 parcellation. The clustering analysis of these maps can be found in S17 Fig. The lifespan trajectory of the extended set of energy maps can be found in S19 Fig. atpsynth, ATP synthase complex; BCAA, branch chained amino acid; pdc, pyruvate dehydrogenase complex; mas, malate-aspartate shuttle; gps: glycerol-3-phosphate shuttle; ros detox, detoxification of reactive oxygen species; ros gen, generation of reactive oxygen species; no signaling, nitric oxide signaling; gln-glu cycle, glutamine-glutamate cycle. (PDF) [file pbio.3003619.s016.pdf]

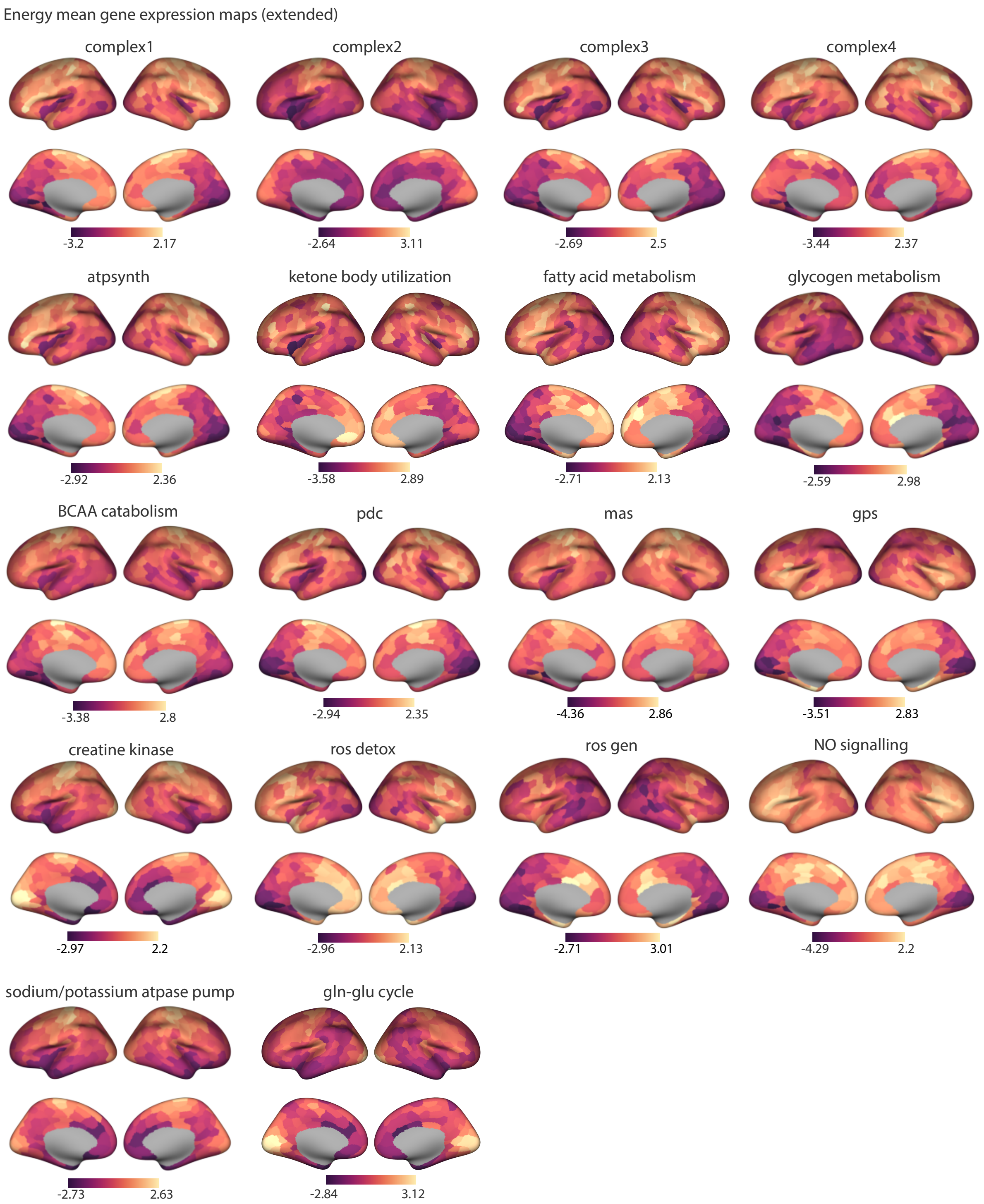

S16 Fig. **Extended set of energy related pathways.** Mean gene expression maps were produced as before (see *Methods*). Color bars represent mean gene expression, z-scored across the 400 cortical regions in the Schaefer-400 parcellation. The clustering analysis of these maps can be found in S17 Fig. The lifespan trajectory of the extended set of energy maps can be found in S19 Fig. atpsynth, ATP synthase complex; BCAA, branch chained amino acid; pdc, pyruvate dehydrogenase complex; mas, malate-aspartate shuttle; gps: glycerol-3-phosphate shuttle; ros detox, detoxification of reactive oxygen species; ros gen, generation of reactive oxygen species; no signaling, nitric oxide signaling; gln-glu cycle, glutamine-glutamate cycle.
