# Supplementary material for: Five energy metabolism pathways show distinct regional distributions and lifespan trajectories in the human brain
Source: PLoS Biol. 2026 Jan 30;24(1):e3003619. doi: 10.1371/journal.pbio.3003619 (PMC12875592; doi:10.1371/journal.pbio.3003619)
Supplement: S17 Fig — (a) Left: Clustering of the complete set of energy maps was carried out using PCA on a matrix containing all pathway mean gene expressions. Axes represent the first and second principal components. Right: hierarchical clustering of extended energy maps. Colorbar represents expression values across the 400 cortical regions in the Schaefer parcellation. (b) Pair-wise correlation among the extended energy maps. Colorbar represents Spearman’s correlation values. Bold edges indicate statistical significance tested against 10 000 spatial nulls and after FDR correction using the Bejamini-Hochberg method for multiple comparisons. atpsynth, ATP synthase complex; BCAA, branch-chained amino acids; pdc, pyruvate dehydrogenase complex; mas, malate-aspartate shuttle; gps: glycerol-3-phosphate shuttle; ros gen, generation of reactive oxygen species; no signaling, nitric oxide signaling; gln-glu cycle, glutamine-glutamate cycle. (PDF) [file pbio.3003619.s017.pdf]

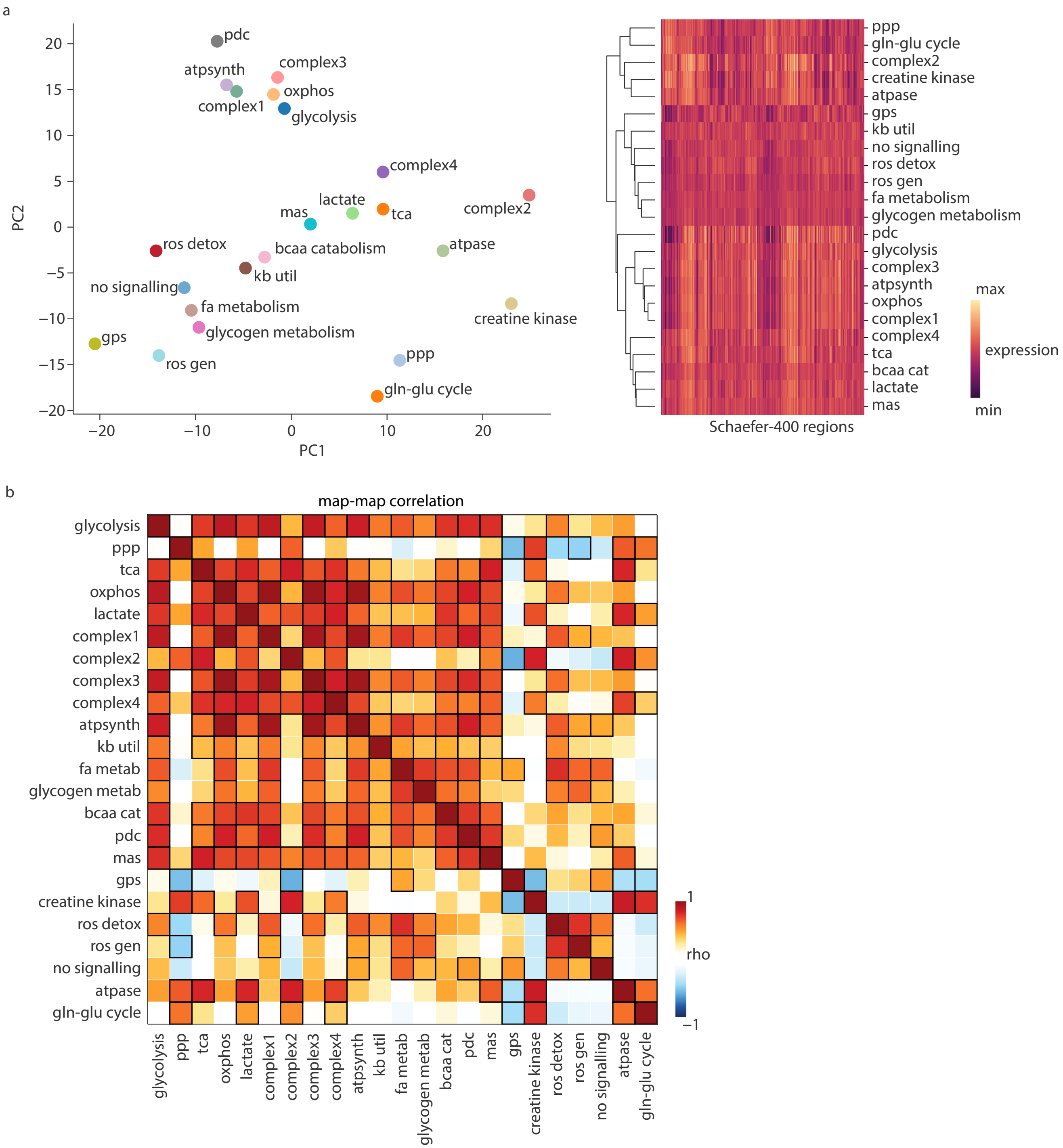


S17 Fig. **Clustering analysis of energy maps.** (a) Left: Clustering of the complete set of energy maps was carried out using PCA on a matrix containing all pathway mean gene expressions. Axes represent the first and second principal components. Right: hierarchical clustering of extended energy maps. Colorbar represents expression values across the 400 cortical regions in the Schaefer parcellation. (b) Pair-wise correlation among the extended energy maps. Colorbar represents Spearman's correlation values. Bold edges indicate statistical significance tested against 10 000 spatial nulls and after FDR correction using the Bejamini-Hochberg method for multiple comparisons. atpsynth, ATP synthase complex; BCAA, branch-chained amino acids; pdc, pyruvate dehydrogenase complex; mas, malate-aspartate shuttle; gps: glycerol-3-phosphate shuttle; ros gen, generation of reactive oxygen species; no signaling, nitric oxide signaling; gln-glu cycle, glutamine-glutamate cycle.
