# Supplementary material for: Five energy metabolism pathways show distinct regional distributions and lifespan trajectories in the human brain
Source: PLoS Biol. 2026 Jan 30;24(1):e3003619. doi: 10.1371/journal.pbio.3003619 (PMC12875592; doi:10.1371/journal.pbio.3003619)
Supplement: S18 Fig — Left: Venn diagram depicts the final number of genes in each mitochondrial complex and their overlap. Right: Heatmap depicts pairwise Spearman’s correlations between mean gene expression maps. Gene sets for each mitochondrial complex were retrieved using GO pathway IDs and mean expression maps where produced as before (see Methods). Asterisks show statistical significance when tested against a distribution of 10 000 correlations produced using spatial autocorrelation preserving permutation test. atpsynth, ATP synthase complex. (PDF) [file pbio.3003619.s018.pdf]

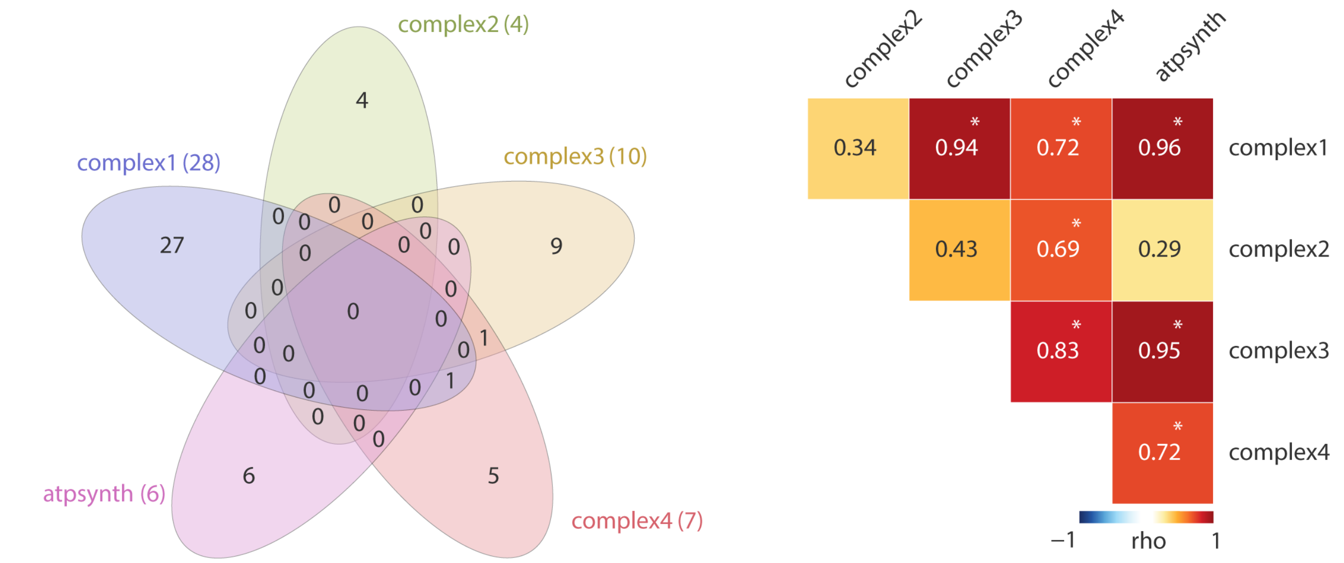

S18 Fig. **Alignment between the components of mitochondrial respiratory chain.** Left: Venn diagram depicts the final number of genes in each mitochondrial complex and their overlap. Right: Heatmap depicts pairwise Spearman's correlations between mean gene expression maps. Gene sets for each mitochondrial complex were retrieved using GO pathway IDs and mean expression maps where produced as before (see *Methods*). Asterisks show statistical significance when tested against a distribution of 10 000 correlations produced using spatial autocorrelation preserving permutation test. atpsynth, ATP synthase complex.
