# Supplementary material for: Five energy metabolism pathways show distinct regional distributions and lifespan trajectories in the human brain
Source: PLoS Biol. 2026 Jan 30;24(1):e3003619. doi: 10.1371/journal.pbio.3003619 (PMC12875592; doi:10.1371/journal.pbio.3003619)
Supplement: S19 Fig — Developmental trajectories of energy metabolism pathways were produced using the BrainSpan RNA-seq expression dataset. For each energy pathway, mean expression was calculated across all genes for each sample and median expression across all samples falling into each age group was calculated. Analysis only included cortical regions. the y-axis represents upper quartile normalized log2(RPKM) expression values. Dots represent individual samples in each age group. Line plot depicts the trajectory of median gene expression. atpsynth, ATP synthase complex; FA, fatty acid; BCAA, branch chained amino acid; pdc, pyruvate dehydrogenase complex; mas, malate-aspartate shuttle; gps: glycerol-3-phosphate shuttle; ros detox: detoxification of reactive oxygen species; ros gen, generation of reactive oxygen species; no signaling, nitric oxide signaling; gln-glu cycle, glutamine-glutamate cycle. (PDF) [file pbio.3003619.s019.pdf]

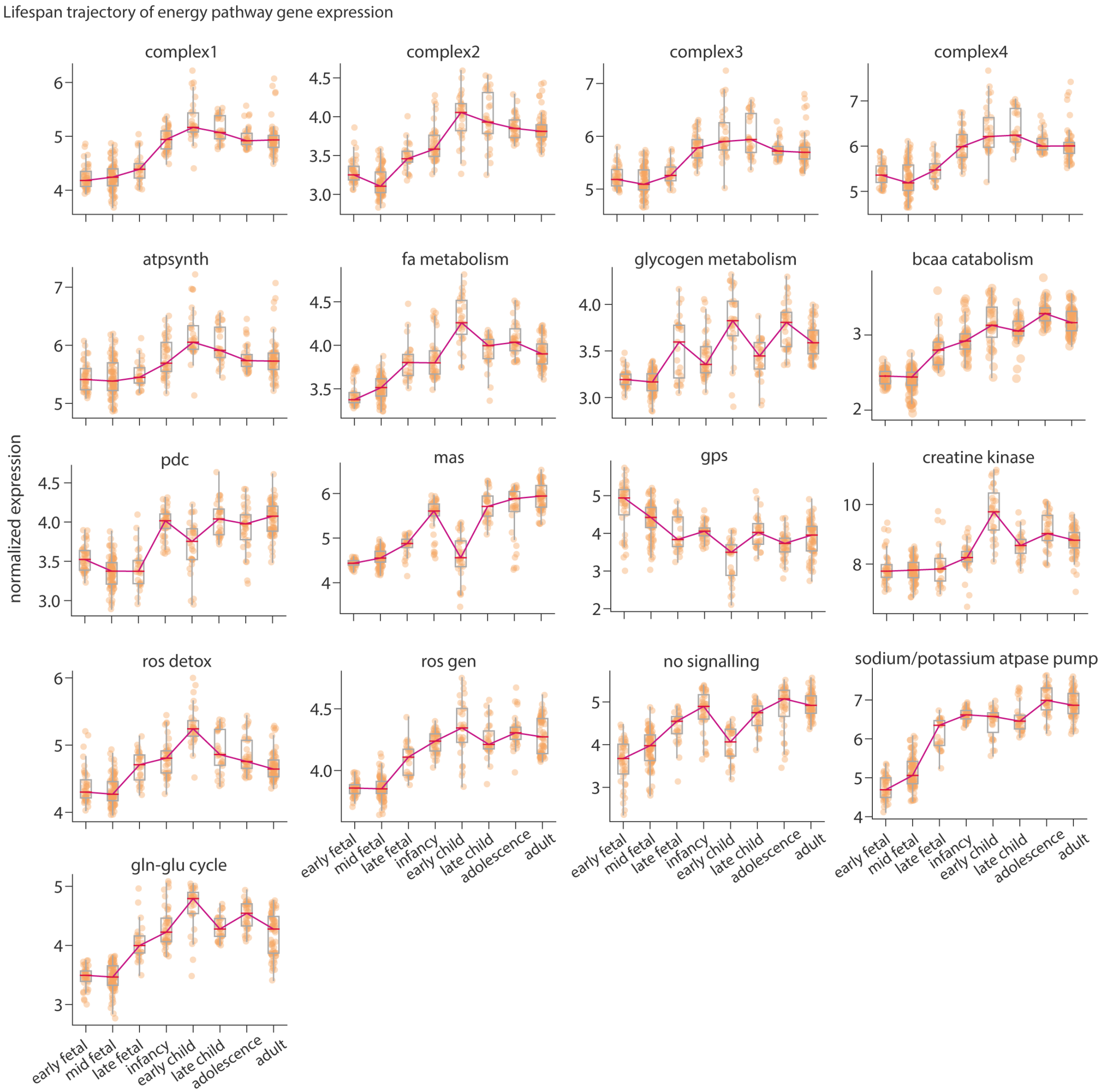

S19 Fig. **Lifespan trajectory of the extended energy metabolism pathways.** Developmental trajectories of energy metabolism pathways were produced using the BrainSpan RNA-seq expression dataset. For each energy pathway, mean expression was calculated across all genes for each sample and median expression across all samples falling into each age group was calculated. Analysis only included cortical regions. the y-axis represents upper quartile normalized $\log_2$(RPKM) expression values. Dots represent individual samples in each age group. Line plot depicts the trajectory of median gene expression. atpsynth, ATP synthase complex; FA, fatty acid; BCAA, branch chained amino acid; pdc, pyruvate dehydrogenase complex; mas, malate-aspartate shuttle; gps: glycerol-3-phosphate shuttle; ros detox: detoxification of reactive oxygen species; ros gen, generation of reactive oxygen species; no signaling, nitric oxide signaling; gln-glu cycle, glutamine-glutamate cycle.
