# Supplementary material for: Five energy metabolism pathways show distinct regional distributions and lifespan trajectories in the human brain
Source: PLoS Biol. 2026 Jan 30;24(1):e3003619. doi: 10.1371/journal.pbio.3003619 (PMC12875592; doi:10.1371/journal.pbio.3003619)
Supplement: S20 Fig — Spatial alignment between the maps were calculated using the Spearman’s correlation and tested against a distribution of 10 000 correlations produced from the spatial permutation nulls. The x-axis represents the PPP mean gene expression. Dots in each scatter plot represent the 400 cortical regions in the Schaefer-400 parcellation. ppp, pentose phosphate pathway. (PDF) [file pbio.3003619.s020.pdf]

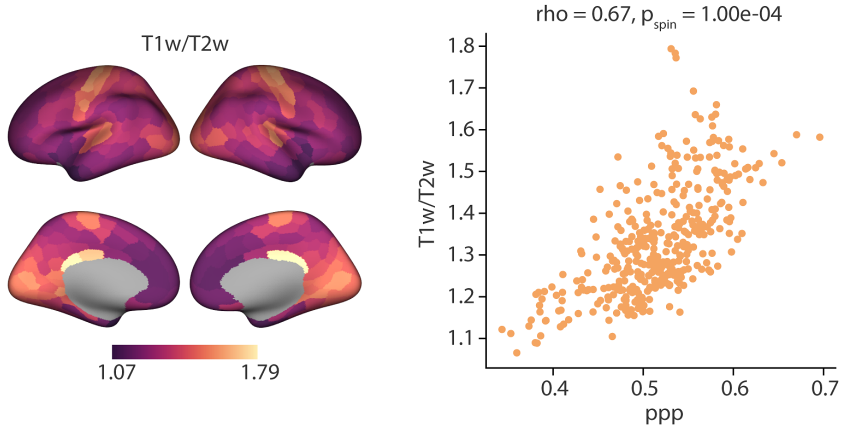

S20 Fig. **Alignment between the PPP and cortical T1w/T2w map.** Spatial alignment between the maps were calculated using the Spearman's correlation and tested against a distribution of 10 000 correlations produced from the spatial permutation nulls. The x-axis represents the PPP mean gene expression. Dots in each scatter plot represent the 400 cortical regions in the Schaefer-400 parcellation. ppp, pentose phosphate pathway.
