# Supplementary material for: Five energy metabolism pathways show distinct regional distributions and lifespan trajectories in the human brain
Source: PLoS Biol. 2026 Jan 30;24(1):e3003619. doi: 10.1371/journal.pbio.3003619 (PMC12875592; doi:10.1371/journal.pbio.3003619)
Supplement: S21 Fig — The differential stability distribution of the final energy pathway genes. Expression data were filtered to have a differential stability value > = 0.1. Dots represent individual genes in each pathway. ppp, pentose phosphate pathway; tca, tricarboxylic acid cycle; oxphos, oxidative phosphorylation; lactate, lactate metabolism and transport. (PDF) [file pbio.3003619.s021.pdf]

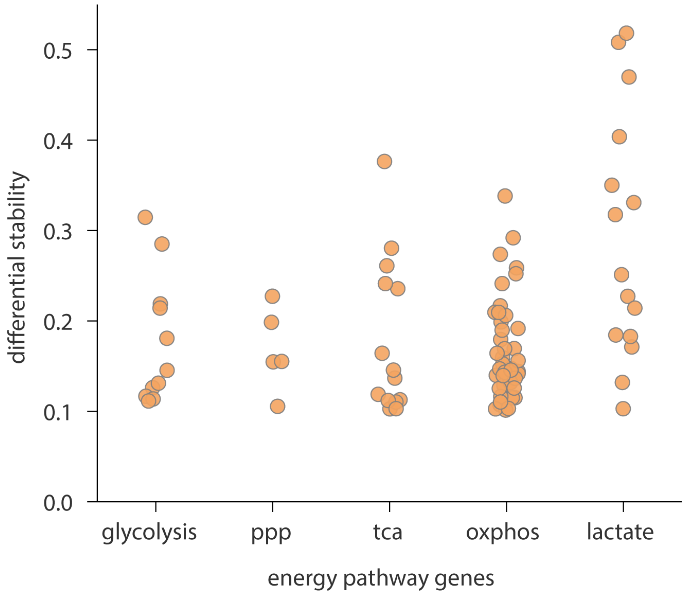

S21 Fig. **Differential stability of energy pathway genes.** The differential stability distribution of the final energy pathway genes. Expression data were filtered to have a differential stability value $>= 0.1$. Dots represent individual genes in each pathway. ppp, pentose phosphate pathway; tca, tricarboxylic acid cycle; oxphos, oxidative phosphorylation; lactate, lactate metabolism and transport.
