# Supplementary material for: Five energy metabolism pathways show distinct regional distributions and lifespan trajectories in the human brain
Source: PLoS Biol. 2026 Jan 30;24(1):e3003619. doi: 10.1371/journal.pbio.3003619 (PMC12875592; doi:10.1371/journal.pbio.3003619)
Supplement: S1 Table — Overview of energy metabolic pathways and their final gene sets included in this study. Gene sets were produced based on GO biological processes and Reactome pathway IDs. Genes annotated in both databases used for the analyses are listed. ppp, pentose phosphate pathway; tca, tricarboxylic acid cycle; oxphos, oxidative phosphorylation; Lactate, lactate metabolism and transport. (PDF) [file pbio.3003619.s022.pdf]

S1 Table. **Energy metabolic pathway gene sets.** Overview of energy metabolic pathways and their final gene sets included in this study. Gene sets were produced based on GO biological processes and Reactome pathway IDs. Genes annotated in both databases used for the analyses are listed. ppp, pentose phosphate pathway; tca, tricarboxylic acid cycle; oxphos, oxidative phosphorylation; Lactate, lactate metabolism and transport.

Pathway	Genes	Pathway IDs
Glycolysis	*ALDOC, ENO1, ENO3, GPI, PFKFB2, PFKL, PFKM, PFKP, PGAM1, PGK1, TPI1*	GO:0006096, R-HSA-70171
PPP	*PGD, PRPS2, RBKS, RPEL1, RPIA*	GO:0006098, R-HSA-71336
TCA	*ACO2, CS, DLST, FH, IDH2, IDH3A, IDH3G, MDH2, OGDH, SDHA, SDHB, SDHD, SUCLA2, SUCLG1*	GO:0006099, R-HSA-71403
OXPHOS	*ATP5F1A, ATP5F1B, ATP5ME, ATP5MF, ATP5PB, ATP5PF, COX4I1, COX6A1, CYC1, CYCS, NDUFA3, NDUFA4, NDUFA6, NDUFA8, NDUFA9, NDUFAB1, NDUFAF1, NDUFB1, NDUFB2, NDUFB3, NDUFB5, NDUFB6, NDUFB8, NDUFB9, NDUFC2, NDUFS1, NDUFS2, NDUFS3, NDUFS5, NDUFS6, NDUFV1, NDUFV2, NDUFV3, SDHA, SDHB, SDHD, UQCR10, UQCR11, UQCRC1, UQCRC2, UQCRFS1, UQCRH, UQCRQ*	GO:0019646, GO:0046933, R-HSA-611105, R-HSA-163210
Lactate	*EMB, HAGH, HAGHL, LDHA, LDHB, LDHC, LDHD, MRS2, PER2, PFKFB2, PNKD, SLC16A3, SLC16A7, SLC16A8, TIGAR*	GO:0006089, GO:0015727
