# Supplementary material for: Five energy metabolism pathways show distinct regional distributions and lifespan trajectories in the human brain
Source: PLoS Biol. 2026 Jan 30;24(1):e3003619. doi: 10.1371/journal.pbio.3003619 (PMC12875592; doi:10.1371/journal.pbio.3003619)
Supplement: S2 Table — Visual regions in the Glasser atlas. Delineations were defined according to the supplementary neuroanatomical results from [59] and https://neuroimaging-core-docs.readthedocs.io/en/latest/pages/atlases.html. (PDF) [file pbio.3003619.s023.pdf]

S2 Table. **Visual ROIs.** Visual regions in the Glasser atlas. Delineations were defined according to the supplementary neuroanatomical results from Glasser et al. [1] and https://neuroimaging-core-docs.readthedocs.io/en/latest/pages/atlases.html.

Label	Region abb.	Region name	Delineation
1	V1	Primary Visual Cortex	Primary Visual
2	MST	Medial Superior Temporal Area	MT+ Complex and Neighboring Visual Areas
3	V6	Sixth Visual Area	Dorsal Stream Visual
4	V2	Second Visual Area	Early Visual
5	V3	Third Visual Area	Early Visual
6	V4	Fourth Visual Area	Early Visual
7	V8	Eighth Visual Area	Ventral Stream Visual
13	V3A	Visual Area V3A	Dorsal Stream Visual
16	V7	Seventh Visual Area	Dorsal Stream Visual
17	IPS1	IntraParietal Sulcus Area 1	Dorsal Stream Visual
18	FFC	Fusiform Face Complex	Ventral Stream Visual
19	V3B	Visual Area V3B	Dorsal Stream Visual
20	LO1	Lateral Occipital Area 1	MT+ Complex and Neighboring Visual Areas
21	LO2	Lateral Occipital Area 2	MT+ Complex and Neighboring Visual Areas
22	PIT	Posterior InferoTemporal complex	Ventral Stream Visual
23	MT	Middle Temporal Area	MT+ Complex and Neighboring Visual Areas
138	PH	Area PH	MT+ Complex and Neighboring Visual Areas
152	V6A	Visual Area V6A	Dorsal Stream Visual
153	VMV1	VentroMedial Visual Area 1	Ventral Stream Visual
154	VMV3	VentroMedial Visual Area 3	Ventral Stream Visual
156	V4t	Visual Area V4t	MT+ Complex and Neighboring Visual Areas
157	FST	Area FST	MT+ Complex and Neighboring Visual Areas
158	V3CD	Visual Area 3 c/d	MT+ Complex and Neighboring Visual Areas
159	LO3	Lateral Occipital Area 3	MT+ Complex and Neighboring Visual Areas
160	VMV2	VentroMedial Visual Area 2	Ventral Stream Visual
163	VVC	Ventral Visual Complex	Ventral Stream Visual

References

1. Glasser MF, Coalson TS, Robinson EC, Hacker CD, Harwell J, Yacoub E, et al. A multi-modal parcellation of human cerebral cortex. Nature. 2016 Aug;536(7615):171-8.
