# Supplementary material for: Five energy metabolism pathways show distinct regional distributions and lifespan trajectories in the human brain
Source: PLoS Biol. 2026 Jan 30;24(1):e3003619. doi: 10.1371/journal.pbio.3003619 (PMC12875592; doi:10.1371/journal.pbio.3003619)
Supplement: S3 Table — Individual cell-type markers used to produce cortical maps for inhibitory and excitatory neuronal subtypes. Marker genes were obtained from [93,184]. (PDF) [file pbio.3003619.s024.pdf]

S3 Table. **Cell-type marker genes.** Individual cell-type markers used to produce cortical maps for inhibitory and excitatory neuronal subtypes. Marker genes were obtained from Kang et al. [1] and Hodge et al. [2].

Cell type	Marker genes
Pan GABAergic	*GAD1, GAD2*
Parvalbumin+	*PVALB*
Somatostatin+	*SST*
Calbindin+	*CALB1, CALB2*
VIP+	*VIP*
Layer 1 Excitatory	*RELN*
Layer 2-4 Excitatory	*CUX1, UNC5D*
Layer 4 Excitatory	*RORB*
Layer 5 Excitatory	*BCL11B, ETV1, FEZF2*
Layer 6 Excitatory	*FOXP2, NTSR1, SOX5, SSTR2, TBR1, TLE4, ZFPM2*
Betz Cell	*ASGR2*

References

1. Kang HJ, Kawasawa YI, Cheng F, Zhu Y, Xu X, Li M, et al. Spatio-temporal transcriptome of the human brain. Nature. 2011 Oct;478(7370):483-9.
2. Hodge RD, Bakken TE, Miller JA, Smith KA, Barkan ER, Graybuck LT, et al. Conserved cell types with divergent features in human versus mouse cortex. Nature. 2019 Sep;573(7772):61-8.
