# Supplementary material for: Five energy metabolism pathways show distinct regional distributions and lifespan trajectories in the human brain
Source: PLoS Biol. 2026 Jan 30;24(1):e3003619. doi: 10.1371/journal.pbio.3003619 (PMC12875592; doi:10.1371/journal.pbio.3003619)
Supplement: S4 Table — RNA-sequencing data (Genecode v10 summarized to genes) were downloaded from the BrainSpan database (https://www.brainspan.org/static/download.html/). Samples were binned into eight major developmental stages [93]. Last column shows number of cortical samples for each age group used in the analysis. pcw, post conception weeks; mos, months; yrs, years. (PDF) [file pbio.3003619.s025.pdf]

S4 Table. **Age groups for BrainSpan RNA-seq samples.** RNA-sequencing data (Genecode v10 summarized to genes) were downloaded from the BrainSpan database (https://www.brainspan.org/static/download.html/). Samples were binned into eight major developmental stages [1]. Last column shows number of cortical samples for each age group used in the analysis. pcw, post conception weeks; mos, months; yrs, years.

Age group	Age	Num. samples
early fetal	8 pcw, 9 pcw, 12 pcw	40
mid fetal	13 pcw, 16 pcw, 17 pcw, 19 pcw, 21 pcw	85
late fetal	24 pcw, 25 pcw, 26 pcw, 35 pcw, 37 pcw	27
infancy	4 mos, 10 mos, 1 yrs	41
early childhood	2 yrs, 3 yrs, 4 yrs	30
late childhood	8 yrs, 11 yrs	30
adolescence	13 yrs, 15 yrs, 18 yrs, 19 yrs	36
adulthood	21 yrs, 23 yrs, 30 yrs, 36 yrs, 37 yrs, 40 yrs	63

References

1. Kang HJ, Kawasawa YI, Cheng F, Zhu Y, Xu X, Li M, et al. Spatio-temporal transcriptome of the human brain. Nature. 2011 Oct;478(7370):483-9.
