# Supplementary material for: Five energy metabolism pathways show distinct regional distributions and lifespan trajectories in the human brain
Source: PLoS Biol. 2026 Jan 30;24(1):e3003619. doi: 10.1371/journal.pbio.3003619 (PMC12875592; doi:10.1371/journal.pbio.3003619)
Supplement: S5 Table — Energy metabolism pathway gene sets used in lifespan trajectory analysis. Note that the same GO biological processes and Reactome pathway IDs were used across all analysis and the difference in final gene sets for each pathway is the result of different gene data availability in AHBA and BrainSpan datasets. PPP, pentose phosphate pathway; TCA, tricarboxylic acid cycle; OXPHOS, oxidative phosphorylation; Lactate, lactate metabolism and transport; Ketone Body, ketone body utilization. (PDF) [file pbio.3003619.s026.pdf]

S5 Table. **BrainSpan energy pathway gene sets.** Energy metabolism pathway gene sets used in lifespan trajectory analysis. Note that the same GO biological processes and Reactome pathway IDs were used across all analysis and the difference in final gene sets for each pathway is the result of different gene data availability in AHBA and BrainSpan datasets. PPP, pentose phosphate pathway; TCA, tricarboxylic acid cycle; OXPHOS, oxidative phosphorylation; Lactate, lactate metabolism and transport; Ketone Body, ketone body utilization.

Pathway	Genes	Pathway IDs
Glycolysis	*PFKP, PRKACA, ENO1, PGK1, GPI, GCK, ENO3, ALDOC, GAPDH, TPI1, ENO2, PPP2CA, PFKFB2, PFKL, ALDOA, PFKM, ADPGK, PGAM2, PGAM1, BPGM, ENO4*	GO:0006096, R-HSA-70171
PPP	*DERA, PRPS2, PGLS, PGD, RPIA, G6PD, TKT, RBKS, TALDO1, SHPK, RPE*	GO:0006098, R-HSA-71336
TCA	*CS, IDH3G, SDHA, FH, ACO2, IDH3B, OGDH, NNT, SDHB, DLST, SUCLA2, SDHC, MDH2, SUCLG1, IDH3A, SUCLG2, IDH2, SDHD*	GO:0006099, R-HSA-71403
OXPHOS	*NDUFAB1, UQCRC1, NDUFS1, NDUFB4, SDHA, NDUFB2, ATP5D, NDUFB7, NDUFC1, NDUFS8, ATP5B, COX6A1, NDUFS7, COX7A2L, ATP5F1, SDHB, NDUFB3, NDUFA8, ATP5E, NDUFA1, COX6B1, COX7C, UQCR11, NDUFA5, NDUFA10, COX4I1, COX7B, NDUFA2, COX5B, NDUFB5, NDUFAF1, NDUFA9, UQCRC2, NDUFB10, SDHC, NDUFS6, NDUFB9, NDUFC2, ATP5A1, ATP5J, UQCRB, NDUFS2, NDUFV3, NDUFS4, UQCRQ, COX6C, NDUFB6, ATP5C1, NDUFB8, ATP5L, NDUFA7, NDUFV1, ATP5H, NDUFS5, ATP5I, UQCRFS1, NDUFA3, CYCS, UQCRH, COX8A, NDUFV2, COX5A, CYC1, NDUFB1, UQCR10, NDUFA6, NDUFA4, SDHD, NDUFS3, ATP5J2, ATP5O*	GO:0019646, GO:0046933, R-HSA-611105, R-HSA-163210
Lactate	*HAGH, ACACB, SLC16A8, HIF1A, HAGHL, LDHB, PARK7, PFKFB2, MRS2, PNKD, PER2, LDHA, SLC37A4, TP53, SLC16A3, SLC16A1, LDHD*	GO:0006089, GO:0015727
Ketone body	*ACAT1, OXCT1*	GO:0046952, R-HSA-77108
