# Supplementary material for: Five energy metabolism pathways show distinct regional distributions and lifespan trajectories in the human brain
Source: PLoS Biol. 2026 Jan 30;24(1):e3003619. doi: 10.1371/journal.pbio.3003619 (PMC12875592; doi:10.1371/journal.pbio.3003619)
Supplement: S6 Table — As before, gene sets were produced based on GO biological processes and Reactome pathway IDs. Genes annotated in both databases used for the analyses are listed. PPP, pentose phosphate pathway; TCA, tricarboxylic acid cycle; OXPHOS, oxidative phosphorylation; Lactate, lactate metabolism and transport. (PDF) [file pbio.3003619.s027.pdf]

S6 Table. **Energy metabolic pathway gene sets without the differential stability threshold.** As before, gene sets were produced based on GO biological processes and Reactome pathway IDs. Genes annotated in both databases used for the analyses are listed. PPP, pentose phosphate pathway; TCA, tricarboxylic acid cycle; OXPHOS, oxidative phosphorylation; Lactate, lactate metabolism and transport.

Pathway	Genes
Glycolysis	*ADPGK, ALDOA, ALDOC, BPGM, ENO1, ENO2, ENO3, ENO4, GAPDH, GAPDHS, GCK, GPI, PFKFB2, PFKL, PFKM, PFKP, PGAM1, PGAM2, PGK1, PPP2CA, PRKACA, TPI1*
PPP	*DERA, G6PD, PGD, PGLS, PRPS2, RBKS, RPE, RPEL1, RPIA, SHPK, TALDO1, TKT*
TCA	*ACO2, CS, DLST, FH, IDH2, IDH3A, IDH3B, IDH3G, MDH2, NNT, OGDH, SDHA, SDHB, SDHC, SDHD, SUCLA2, SUCLG1, SUCLG2*
OXPHOS	*ATP5F1A, ATP5F1B, ATP5F1C, ATP5F1D, ATP5ME, ATP5MF, ATP5MG, ATP5PB, ATP5PD, ATP5PF, ATP5PO, COX4I1, COX5A, COX5B, COX6A1, COX6B1, COX6C, COX7A2L, COX7B, COX7C, COX8A, CYC1, CYCS, NDUFA1, NDUFA10, NDUFA2, NDUFA3, NDUFA4, NDUFA5, NDUFA6, NDUFA7, NDUFA8, NDUFA9, NDUFAB1, NDUFAF1, NDUFB1, NDUFB10, NDUFB2, NDUFB3, NDUFB4, NDUFB5, NDUFB6, NDUFB7, NDUFB8, NDUFB9, NDUFC1, NDUFC2, NDUFS1, NDUFS2, NDUFS3, NDUFS4, NDUFS5, NDUFS6, NDUFS7, NDUFS8, NDUFV1, NDUFV2, NDUFV3, SDHA, SDHB, SDHC, SDHD, UQCR10, UQCR11, UQCRB, UQCRC1, UQCRC2, UQCRFS1, UQCRH, UQCRQ*
Lactate	*ACACB, EMB, GATD1, HAGH, HAGHL, HIF1A, LDHA, LDHAL6A, LDHB, LDHC, LDHD, MRS2, PARK7, PER2, PFKFB2, PNKD, SLC16A1, SLC16A3, SLC16A7, SLC16A8, SLC37A4, SLC5A12, TIGAR*
