# Supplementary material for: Five energy metabolism pathways show distinct regional distributions and lifespan trajectories in the human brain
Source: PLoS Biol. 2026 Jan 30;24(1):e3003619. doi: 10.1371/journal.pbio.3003619 (PMC12875592; doi:10.1371/journal.pbio.3003619)
Supplement: S7 Table — Genes not available in the AHBA, as well as genes not fulfilling the differential stability threshold were excluded from the OXPHOS gene expression matrix. (PDF) [file pbio.3003619.s028.pdf]

S7 Table. **Excluded genes in the OXPHOS map broken down by individual complexes.** Genes not available in the AHBA, as well as genes not fulfilling the differential stability threshold were excluded from the OXPHOS gene expression matrix.

MT complex	Excluded genes
complex1	*MT-ND1, MT-ND2, MT-ND3, MT-ND4, MT-ND5, MT-ND6, NDUFC1, NDUFA1, NDUFA7, NDUFB7, NDUFA5, NDUFA10, NDUFS4, NDUFB10, NDUFS7, NDUFA2, NDUFS8, NDUFB4*
complex2	*SDHC*
complex3	*MT-CYB, UQCRB*
complex4	*MT-CO1, MT-CO2, MT-CO3, COX5B, COX7B, COX7A2L, COX8A, COX6C, COX7C, COX5A, COX6B1*
atpsynth	*MT-ATP8, MT-ATP6, ATP5PO, ATP5F1C, ATP5F1E, ATP5F1D, ATP5PD, ATP5MG*
