# Supplementary material for: Five energy metabolism pathways show distinct regional distributions and lifespan trajectories in the human brain
Source: PLoS Biol. 2026 Jan 30;24(1):e3003619. doi: 10.1371/journal.pbio.3003619 (PMC12875592; doi:10.1371/journal.pbio.3003619)
Supplement: S8 Table — Microarray data were downloaded from the BrainSpan database (https://www.brainspan.org/static/download.html/) Samples were binned into eight major developmental stages [93]. Last column shows number of cortical samples for each age group used in the analysis. pcw, Post conception weeks; mos, months; yrs, years. (PDF) [file pbio.3003619.s029.pdf]

S8 Table. **Age groups for BrainSpan microarray samples.** Microarray data were downloaded from the BrainSpan database (https://www.brainspan.org/static/download.html/) Samples were binned into eight major developmental stages [1]. Last column shows number of cortical samples for each age group used in the analysis. pcw, Post conception weeks; mos, months; yrs, years.

Age group	Age	Num. samples
early fetal	8 pcw, 9 pcw, 12 pcw	42
mid fetal	13 pcw, 16 pcw, 17 pcw, 19 pcw, 21 pcw	95
late fetal	24 pcw, 25 pcw, 26 pcw	16
infancy	4 mos, 10 mos, 1 yrs	31
early childhood	2 yrs, 3 yrs, 4 yrs	40
late childhood	8 yrs	11
adolescence	13 yrs, 15 yrs, 18 yrs	33
adulthood	21 yrs, 23 yrs, 30 yrs, 36 yrs, 37 yrs, 40 yrs	66

References

1. Kang HJ, Kawasawa YI, Cheng F, Zhu Y, Xu X, Li M, et al. Spatio-temporal transcriptome of the human brain. Nature. 2011 Oct;478(7370):483-9.
