# Supplementary material for: Five energy metabolism pathways show distinct regional distributions and lifespan trajectories in the human brain
Source: PLoS Biol. 2026 Jan 30;24(1):e3003619. doi: 10.1371/journal.pbio.3003619 (PMC12875592; doi:10.1371/journal.pbio.3003619)
Supplement: S9 Table — As before, gene sets were produced based on GO biological processes and Reactome pathway IDs. When available in both databases, consensus genes used to create the energy mean expression maps are listed. PPP, pentose phosphate pathway; TCA, tricarboxylic acid cycle; OXPHOS, oxidative phosphorylation; Lactate, lactate metabolism and transport. (PDF) [file pbio.3003619.s030.pdf]

S9 Table. **Gene sets for the extended set of energy pathways.** As before, gene sets were produced based on GO biological processes and Reactome pathway IDs. When available in both databases, consensus genes used to create the energy mean expression maps are listed. PPP, pentose phosphate pathway; TCA, tricarboxylic acid cycle; OXPHOS, oxidative phosphorylation; Lactate, lactate metabolism and transport.

Pathway	Genes	Pathway IDs
Complex 1	*DLD, DNAJC15, ISCU, NDUFA3, NDUFA4, NDUFA6, NDUFA8, NDUFA9, NDUFAB1, NDUFAF1, NDUFB1, NDUFB2, NDUFB3, NDUFB5, NDUFB6, NDUFB8, NDUFB9, NDUFC2, NDUFS1, NDUFS2, NDUFS3, NDUFS5, NDUFS6, NDUFV1, NDUFV2, NDUFV3, PINK1, SNCA*	GO:0006120
Complex 2	*SDHA, SDHAF2, SDHB, SDHD*	GO:0006121
Complex 3	*CYC1, CYCS, UQCC3, UQCR10, UQCR11, UQCRC1, UQCRC2, UQCRFS1, UQCRH, UQCRQ*	GO:0006122
Complex 4	*AFG1L, COX4I1, COX6A1, COX6A2, COX7A1, CYCS, NDUFA4*	GO:0006123
ATP synthase	*ATP5F1A, ATP5F1B, ATP5ME, ATP5MF, ATP5PB, ATP5PF*	GO:0046933, R-HSA-163210
Ketone body	*BDH1, BDH2, OXCT1, OXCT2*	GO:0046952, R-HSA-77108
FA metabolism	*ACAA1, ACAA2, ACADL, ACOT2, ACOT8, ACOX2, ACOX3, ACSF2, ACSF3, ACSL3, ACSL4, ACSL6, AKR1C3, ALOX5, ALOX5AP, CPT1A, CROT, CYP1B1, CYP2U1, DBI, ECHS1, ELOVL1, ELOVL4, ELOVL7, FAAH2, FADS1, FADS2, GGT1, GGT5, GPX4, HACD1, HACD2, HACD4, HACL1, HADH, HPGD, HSD17B12, HSD17B4, HSD17B8, MECR, NDUFAB1, NUDT7, PCCA, PCCB, PECR, PHYH, PLA2G4A, PPARD, PRKAA2, PRKAB2, PRKAG2, PTGES, PTGIS, PTGR1, PTGR2, PTGS2, SLC25A17, SLC27A2, SLC27A3, THEM4*	GO:0006631, R-HSA-8978868
Glycogen metabolism	*AGL, EPM2A, GBE1, GYG2, NHLRC1, PHKA2, PYGL, UGP2*	GO:0005977, R-HSA-8982491
BCAA catabolism	*ALDH6A1, AUH, BCAT1, BCKDK, HIBADH, MCCC1, MCCC2*	GO:0009083, R-HSA-70895
PDC	*DLD, PDHA1, PDHB*	GO:0045254
MAS	*GOT1, GOT2, MDH2, SLC25A12, SLC25A13, SLC25A18, SLC25A22*	GO:0043490, R-HSA-9856872
GPS	*GPD1, GPD1L, GPD2*	GO:0006127, R-HSA-188467
Creatine kinase	*CKMT1A, CKMT1B, CKMT2*	GO:0004111
ROS detox	*ATOX1, CCS, CYBB, CYCS, GPX3, GPX7, GSTP1, NUDT2, PRDX1, PRDX2, PRDX3, PRDX6, SOD1, SOD2, SOD3, TXN, TXN2, TXNRD1, TXNRD2*	R-HSA-3299685
ROS generation	*ABCB7, ABCD2, ALOX5, ARG2, CFLAR, CYBB, DMD, HVCN1, LIPA, MAOB, PLCG2, SNCA, SOD1, SOD2, SPHK2, TLR4*	GO:1903409
NO signalling	*GUCY1A1, GUCY1A2, ITPR1, KCNMB1, KCNMB2, KCNMB4, NOS1, NOS2, PDE10A, PDE11A, PDE1A, PDE1B, PDE2A, PRKG2*	R-HSA-392154
ATPase pump	*ATP1A1, ATP1A3, ATP1A4, ATP1B1, ATP1B3, FXYD1*	GO:0005890
Gln-Glu cycle	*GLS2, SLC1A6, SLC1A7, SLC38A1, SLC38A2, SLC38A3, SLC38A5, SLC38A7*	-
