# Supplementary material for: Five energy metabolism pathways show distinct regional distributions and lifespan trajectories in the human brain
Source: PLoS Biol. 2026 Jan 30;24(1):e3003619. doi: 10.1371/journal.pbio.3003619 (PMC12875592; doi:10.1371/journal.pbio.3003619)
Supplement: Attachment — Submitted filename: pourmajidian_response_to_reviewers.pdf [file pbio.3003619.s032.pdf]

Dear Dr. Hart and Reviewers,

We appreciate the valuable feedback provided on our initial submission. In response to the reviewers' suggestions, we have carried out several new analyses and made substantial revisions to the manuscript. Below, reviewer comments appear in bold font, with our responses in regular font. Revisions added to the manuscript are provided in blue. Key changes include:

- Added analyses and interpretation regarding individual cell type markers and their spatial alignment with energy metabolism maps.

- Added sensitivity analysis using the first principal component of pathway gene expression.

- Included tables of gene sets used for extended sets of energy metabolism pathway maps and gene sets used in sensitivity analysis without the differential stability threshold.

- Extended discussion and interpretation regarding the weak correlation between OXPHOS gene expression and $CMR_{O2}$.

- Added clarification on the exclusion of mitochondrially-encoded genes in energy metabolism pathway expression.

- Included clarification of the methodological details regarding the cell type-specific and laminar organization maps.

Sincerely,
Moohebat Pourmajidian, Bratislav Misic and Alain Dagher

**Reviewer #1**
**Anna Sophia Monzel:**

**The manuscript by Pourmajidian et al. maps five major energy metabolism pathways (glycolysis, TCA, OxPhos, PPP, and lactate, which they term "energy maps") across human cortical regions at the transcriptomics level, linking them to structural, functional, and developmental features. Using curated gene sets with the Allen Human Brain Atlas microarray data, they relate spatial gene expression patterns to cortical cytoarchitecture, functional connectivity, cell type composition, laminar organization, and PET/MRI metabolic imaging, and extend analyses to developmental trajectories using BrainSpan RNAseq data. This descriptive study, based entirely on publicly available datasets, reveals distinct regional distributions and lifespan changes for each of the five pathways, highlighting differences between ATP-producing and biosynthetic processes, providing a resource with potential relevance for future neurological and neurodegenerative disease research, once validated in larger cohorts. I have no major concerns with the paper, and I find the study valuable.**

**Strengths:**
**- Whole-brain profiling of key energy pathways using a pathway-based approach, which is more interpretable than differentially expressed genes alone**
**- Inclusion of extended mitochondrial-related pathways (ETC complexes, NADH shuttle, ROS…), which I would have liked to see examined and discussed even further (maybe in a follow-up study)**
**- Linking energy maps to structural, functional, and developmental features**
**- Overall positive correlation with recently published MitoBrainMap data, especially for ETC complexes and mitochondrial respiratory capacity and their OxPhos score**
**- Developmental trajectory analyses (though effect sizes would strengthen)**
**- Thorough and transparent computational methods with clearly documented, well organized code on GitHub**
**- Provides a valuable macro-scale view and method of oxidative and biosynthetic potential (though transcript-level data cannot establish actual functional capacity).**

**Minor points:**

**- Some gene sets are imbalanced (for example, OxPhos is enriched for Complex I genes, only very few CIV genes). It would be useful to see the excluded genes broken down by sub-pathway. The authors addressed this in the end in their sensitivity analysis, where they repeated the analysis with all available genes (which genes were included and found in the dataset?)**

Indeed, the reviewer correctly notes the imbalance in OXPHOS gene representation. This reflects the inherent structural differences among the mitochondrial complexes; Complex I is the largest complex with 45 protein subunits, while Complex II is the smallest with four protein

subunits [1, 2]. We have added this clarification in the results, subsection “Mapping metabolic pathways using gene expression”:

> “It should be noted that the OXPHOS gene set is pre-dominantly populated by mitochondrial complex I genes given its substantially larger size (45 subunits) compared to the other complexes [45]. Additionally, mitochondrially-encoded genes were not included in the AHBA microarray platform and are therefore absent from our pathway maps (Table S7).”

*References:*


*1. Guerrero-Castillo, S. et al. The Assembly Pathway of Mitochondrial Respiratory Chain Complex I. Cell Metab. 25, 128–139 (2017).*

*2. Fullerton, M., McFarland, R., Taylor, R. W. & Alston, C. L. The genetic basis of isolated mitochondrial complex II deficiency. Mol. Genet. Metab. 131, 53–65 (2020).*


Furthermore, regarding the gene sets used in the sensitivity analysis (where we created the main energy pathway maps without applying the differential stability threshold to the AHBA gene expression data), we have now added the complete set of available genes in the AHBA for each pathway in Supplementary Table S6. Importantly, our main findings remained consistent in the sensitivity analysis (Fig. S9), indicating that the spatial patterns may reflect genuine biological organization rather than artifacts of gene selection.

| Pathway | Genes |
|---|---|
| Glycolysis | *ADPGK, ALDOA, ALDOC, BPGM, ENO1, ENO2, ENO3, ENO4, GAPDH, GAPDHS, GCK, GPI, PFKFB2, PFKL, PFKM, PFKP, PGAM1, PGAM2, PGK1, PPP2CA, PRKACA, TPI1* |
| PPP | *DERA, G6PD, PGD, PGLS, PRPS2, RBKS, RPE, RPEL1, RPIA, SHPK, TALDO1, TKT* |
| TCA | *ACO2, CS, DLST, FH, IDH2, IDH3A, IDH3B, IDH3G, MDH2, NNT, OGDH, SDHA, SDHB, SDHC, SDHD, SUCLA2, SUCLG1, SUCLG2* |
| OXPHOS | *ATP5F1A, ATP5F1B, ATP5F1C, ATP5F1D, ATP5ME, ATP5MF, ATP5MG, ATP5PB, ATP5PD, ATP5PF, ATP5PO, COX4I1, COX5A, COX5B, COX6A1, COX6B1, COX6C, COX7A2L, COX7B, COX7C, COX8A, CYC1, CYCS, NDUFA1, NDUFA10, NDUFA2, NDUFA3, NDUFA4, NDUFA5, NDUFA6, NDUFA7, NDUFA8, NDUFA9, NDUFAB1, NDUFAF1, NDUFB1, NDUFB10, NDUFB2, NDUFB3, NDUFB4, NDUFB5, NDUFB6, NDUFB7, NDUFB8, NDUFB9, NDUFC1, NDUFC2, NDUFS1, NDUFS2, NDUFS3, NDUFS4, NDUFS5, NDUFS6, NDUFS7, NDUFS8, NDUFV1, NDUFV2, NDUFV3, SDHA, SDHB, SDHC, SDHD, UQCR10, UQCR11, UQCRB, UQCRC1, UQCRC2, UQCRFS1, UQCRH, UQCRQ* |
| Lactate | *ACACB, EMB, GATD1, HAGH, HAGHL, HIF1A, LDHA, LDHAL6A, LDHB, LDHC, LDHD, MRS2, PARK7, PER2, PFKFB2, PNKD, SLC16A1, SLC16A3, SLC16A7, SLC16A8, SLC37A4, SLC5A12, TIGAR* |

“TABLE S6. Energy metabolic pathway gene sets without the differential stability threshold. As before, gene sets were produced based on GO biological processes and

Reactome pathway IDs. Genes annotated in both databases used for the analyses are listed. PPP, pentose phosphate pathway; TCA, tricarboxylic acid cycle; OXPHOS, oxidative phosphorylation; Lactate, lactate metabolism and transport."

Genes excluded from the OXPHOS gene expression map are shown in Supplementary Table S7 for each individual mitochondrial complex. As shown in this table, some excluded genes were mitochondrial DNA-encoded subunits (e.g., *MT-ND1, MT-ND5, MT-CO1*) that are not available in the AHBA dataset, while some genes were excluded because they did not meet the differential stability threshold.

| MT complex | Excluded genes |
|---|---|
| complex1 | *MT-ND1, MT-ND2, MT-ND3, MT-ND4, MT-ND5, MT-ND6, NDUFC1, NDUFA1, NDUFA7, NDUFB7, NDUFA5, NDUFA10, NDUFS4, NDUFB10, NDUFS7, NDUFA2, NDUFS8, NDUFB4* |
| complex2 | *SDHC* |
| complex3 | *MT-CYB, UQCRB* |
| complex4 | *MT-CO1, MT-CO2, MT-CO3, COX5B, COX7B, COX7A2L, COX8A, COX6C, COX7C, COX5A, COX6B1* |
| atpsynth | *MT-ATP8, MT-ATP6, ATP5PO, ATP5F1C, ATP5F1E, ATP5F1D, ATP5PD, ATP5MG* |

"TABLE S7. Excluded genes in the OXPHOS map broken down by individual complexes. Genes not available in the AHBA, as well as genes not fulfilling the differential stability threshold were excluded from the OXPHOS gene expression matrix."

Furthermore, all original gene sets retrieved from Gene Ontology and Reactome databases are available on our GitHub repository (files: go_genesets.xlsx and reactome_genesets.xlsx). We have also mentioned the availability of these gene sets in the Methods, subsection "Energy pathway gene sets":

"Pathway gene sets retrieved from GO and Reactome databases are provided on our GitHub repository (https://github.com/netneurolab/pourmajidian_metabolism-genes/)."

**- Mitochondrial DNA-encoded transcripts are missing despite their importance for brain energetics. However, those transcripts might not have been available in the AHBA dataset in first place as they are often excluded. Clarification on whether this is due to dataset limitations vs QC exclusions would be helpful.**

We thank the reviewer for this important observation. Mitochondrially-encoded genes (e.g., *MT-ND1, MT-CO1, MT-CYB*) were not probed in the Allen Human Brain Atlas microarray platform and therefore were not included in our pathway gene expression maps. We have now

noted this limitation in the results, subsection “Mapping metabolic pathways using gene expression”:

> “It should be noted that the OXPHOS gene set is predominantly populated by mitochondrial complex I genes due to its substantially larger size (45 subunits) compared to other complexes (Supplementary Table S6). Additionally, mitochondrially-encoded genes are not included in the AHBA microarray platform and are therefore absent from our pathway maps.”

And in the limitations section of the discussion:

> “Additionally, some genes involved in energy metabolic pathways were unavailable or excluded based on quality control criteria (e.g., mitochondrially-encoded genes that encode critical subunits of the electron transport chain were not probed in the AHBA and may exhibit distinct spatial patterns from nuclear-encoded genes due to different regulatory mechanisms).”

**- I could not find gene lists for additional pathways (FAO, CI, CII,…), they would be useful and also needed for future interpretations**

Gene sets for all extended energy pathways have been added in Supplementary Table S9.

The complete original gene sets retrieved from Gene Ontology and Reactome databases are also available in our GitHub repository (files: go_genesets.xlsx and reactome_genesets.xlsx).

| Pathway | Genes | Pathway IDs |
|---|---|---|
| Complex 1 | *DLD, DNAJC15, ISCU, NDUFA3, NDUFA4, NDUFA6, NDUFA8, NDUFA9, NDUFAB1, NDUFAF1, NDUFB1, NDUFB2, NDUFB3, NDUFB5, NDUFB6, NDUFB8, NDUFB9, NDUFC2, NDUFS1, NDUFS2, NDUFS3, NDUFS5, NDUFS6, NDUFV1, NDUFV2, NDUFV3, PINK1, SNCA* | GO:0006120 |
| Complex 2 | *SDHA, SDHAF2, SDHB, SDHD* | GO:0006121 |
| Complex 3 | *CYC1, CYCS, UQCC3, UQCR10, UQCR11, UQCRC1, UQCRC2, UQCRFS1, UQCRH, UQCRQ* | GO:0006122 |
| Complex 4 | *AFG1L, COX4I1, COX6A1, COX6A2, COX7A1, CYCS, NDUFA4* | GO:0006123 |
| ATP synthase | *ATP5F1A, ATP5F1B, ATP5ME, ATP5MF, ATP5PB, ATP5PF* | GO:0046933, R-HSA-163210 |
| Ketone body | *BDH1, BDH2, OXCT1, OXCT2* | GO:0046952, R-HSA-77108 |
| FA metabolism | *ACAA1, ACAA2, ACADL, ACOT2, ACOT8, ACOX2, ACOX3, ACSF2, ACSF3, ACSL3, ACSL4, ACSL6, AKR1C3, ALOX5, ALOX5AP, CPT1A, CROT, CYP1B1, CYP2U1, DBI, ECHS1, ELOVL1, ELOVL4, ELOVL7, FAAH2, FADS1, FADS2, GGT1, GGT5, GPX4, HACD1, HACD2, HACD4, HACL1, HADH, HPGD, HSD17B12, HSD17B4, HSD17B8, MECR, NDUFAB1, NUDT7, PCCA, PCCB, PECR, PHYH, PLA2G4A, PPARD, PRKAA2, PRKAB2, PRKAG2, PTGES, PTGIS, PTGR1, PTGR2, PTGS2, SLC25A17, SLC27A2, SLC27A3, THEM4* | GO:0006631, R-HSA-8978868 |
| Glycogen metabolism | *AGL, EPM2A, GBE1, GYG2, NHLRC1, PHKA2, PYGL, UGP2* | GO:0005977, R-HSA-8982491 |
| BCAA catabolism | *ALDH6A1, AUH, BCAT1, BCKDK, HIBADH, MCCC1, MCCC2* | GO:0009083, R-HSA-70895 |
| PDC | *DLD, PDHA1, PDHB* | GO:0045254 |
| MAS | *GOT1, GOT2, MDH2, SLC25A12, SLC25A13, SLC25A18, SLC25A22* | GO:0043490, R-HSA-9856872 |
| GPS | *GPD1, GPD1L, GPD2* | GO:0006127, R-HSA-188467 |
| Creatine kinase | *CKMT1A, CKMT1B, CKMT2* | GO:0004111 |
| ROS detox | *ATOX1, CCS, CYBB, CYCS, GPX3, GPX7, GSTP1, NUDT2, PRDX1, PRDX2, PRDX3, PRDX6, SOD1, SOD2, SOD3, TXN, TXN2, TXNRD1, TXNRD2* | R-HSA-3299685 |
| ROS generation | *ABCB7, ABCD2, ALOX5, ARG2, CFLAR, CYBB, DMD, HVCN1, LIPA, MAOB, PLCG2, SNCA, SOD1, SOD2, SPHK2, TLR4* | GO:1903409 |
| NO signalling | *GUCY1A1, GUCY1A2, ITPR1, KCNMB1, KCNMB2, KCNMB4, NOS1, NOS2, PDE10A, PDE11A, PDE1A, PDE1B, PDE2A, PRKG2* | R-HSA-392154 |
| ATPase pump | *ATP1A1, ATP1A3, ATP1A4, ATP1B1, ATP1B3, FXYD1* | GO:0005890 |
| Gln-Glu cycle | *GLS2, SLC1A6, SLC1A7, SLC38A1, SLC38A2, SLC38A3, SLC38A5, SLC38A7* | - |

“TABLE S9. Gene sets for the extended set of energy pathways. As before, gene sets were produced based on GO biological processes and Reactome pathway IDs. When available in both databases, consensus genes used to create the energy mean expression maps are listed. PPP, pentose phosphate pathway; TCA, tricarboxylic acid cycle; OXPHOS, oxidative phosphorylation; Lactate, lactate metabolism and transport.”

**- Some genes are not truly pathway members (eg PRPS2 in the PPP set, which is technically part of nucleotide synthesis)**

The reviewer correctly notes that some genes may not be exclusive pathway members. Indeed, PRPS2 functions in nucleotide synthesis downstream of the PPP, rather than within the core PPP itself. However, to ensure systematic and reproducible pathway definitions, we relied on curated functional annotation databases (Gene Ontology and Reactome). As a robustness measure, we retained only genes annotated as pathway members in both databases.

We acknowledge that alternative curation strategies could yield different gene sets and therefore spatial distribution profiles. However, our sensitivity analyses demonstrate that results remain consistent across different gene set definitions (Fig. S9).

Furthermore, to address the reviewer's concern, we tested the PPP map after excluding *PRPS2* and *RBKS* genes that are members of the nucleotide synthesis pathway. We observe a strong correlation with the original PPP map included in our main analysis (rho = 0.89, $p_{spin}$ = 1.00e-04), indicating that the spatial pattern is robust to the inclusion or exclusion of these overlapping cases.

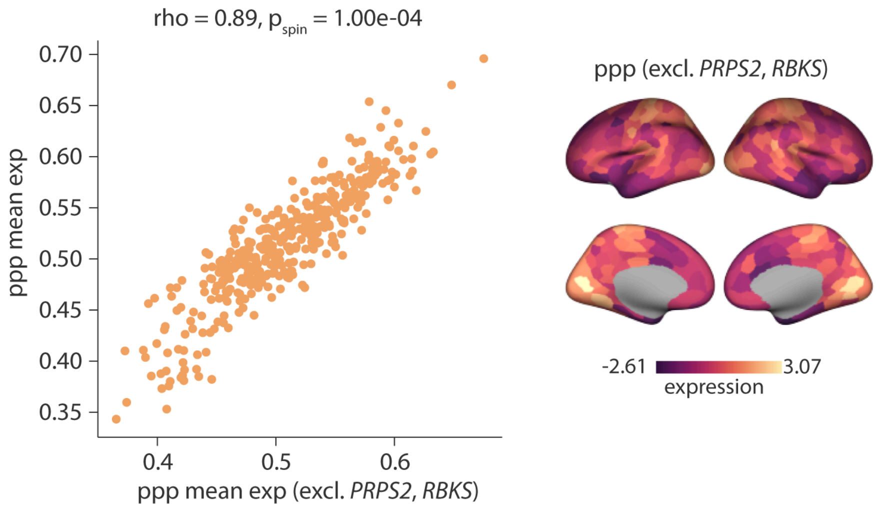


We have clarified that these genes are part of the nucleotide synthesis in the beginning of the results section:

> "However, the PPP gene set predominantly includes genes involved in its anabolic non-oxidative branch (i.e. *RPEL1, RPIA, PRPS2, RBKS*) responsible for the production of 5-carbon sugars and downstream nucleotide synthesis [6, 48]."

**- There is limited dissection of cell-type specific mitochondrial adaptations, and bulk data may mask fine-grained heterogeneity. The authors addressed this sufficiently in their cell type composition correlation in Figure 5 though.**

Indeed, Allen Human Brain Atlas data are obtained from bulk tissue and cannot capture fine-grained heterogeneity in metabolic specialization at the cellular scale. Future research will

benefit from future datasets employing spatial transcriptomics and single-cell RNA-sequencing technologies. In the absence of these data, we used cell-type marker genes as proxy measures, acknowledging the limitations of this approach in the discussion:

> "Finally, transcript levels do not directly correspond to pathway activity or metabolic flux, due to intervening regulatory steps (e.g., transcript splicing, post-translational modifications, protein ubiquitination, phosphorylation and degradation, enzymatic regulation). Therefore, these maps do not account for the post-translational modifications that underlie metabolic activity and specialization [9]. Moreover, the AHBA is obtained from bulk tissue samples where regional gene expression originates from mixed cell populations, limiting the ability to attribute specific energy profiles to a single cell type at this scale. Gene expression as used here could therefore be best viewed as the molecular blueprint of metabolic capacity."

**- The weak OxPhos-CMRO2 correlation is surprising and could be further explored (e.g. testing correlations by individual ETC complex). As the authors point out, transcript abundance does not necessarily reflect functional activity (especially for long-lived mitochondrial proteins), and oxygen consumption may be decoupled from transcription. Also, CMRO2 measurements may underestimate spare respiratory capacity that might be reflected at the mRNA level.**

The reviewer raises several excellent points that further explain the  weak correlation between OXPHOS gene expression and $CMR_{O2}$.

First, Mitochondrial respiratory chain proteins are indeed remarkably long-lived compared to typical cellular proteins, with substantially longer half-lives. This is particularly pronounced in post-mitotic cells such as neurons, where maintaining mitochondrial integrity over extended periods is critical for cellular function [1]. Importantly, core electron transport chain subunits can maintain function even after transcript depletion [2], suggesting an uncoupling of mitochondrial activity from transcription. Therefore, transcript abundance used in our study is likely to  better reflect long-term metabolic capacity rather than acute oxygen consumption rates.

Second, as the reviewer notes, PET-measured $CMR_{O2}$ reflects resting-state oxygen consumption, which may not account for the spare respiratory capacity [3]. This reserve capacity is likely better reflected in gene expression profiles that represent the cell's preparedness for increased metabolic demand rather than moment-to-moment utilization at rest.

Together, these factors explain the discrepancy between gene expression patterns that may better reflect metabolic potential and in vivo resting state oxygen consumption measured by PET imaging. We have now added these points in the discussion:

> "The weak correspondence between OXPHOS gene expression and $CMR_{O2}$ likely reflects several factors. Mitochondrial proteins are among the most long-lived within the

cell, allowing sustained function even after transcript depletion [101, 102]. As a result, OXPHOS gene expression may better capture long-term metabolic capacity rather than acute oxygen use. In addition, $CMR_{O2}$ PET reflects resting oxygen consumption and does not account for spare respiratory capacity, which may be better indexed by transcriptional profiles."

*References:*


*1. Bomba-Warczak, E. & Savas, J. N. Long-lived mitochondrial proteins and why they exist. Trends Cell Biol. 32, 646–654 (2022).*

*2. Krishna, S. et al. Identification of long-lived proteins in the mitochondria reveals increased stability of the electron transport chain. Dev. Cell 56, 2952-2965.e9 (2021).*

*3. Marchetti, P., Fovez, Q., Germain, N., Khamari, R. & Kluza, J. Mitochondrial spare respiratory capacity: Mechanisms, regulation, and significance in non-transformed and cancer cells. FASEB J. 34, 13106–13124 (2020).*


Finally, we tested the spatial alignment of individual ETC complexes with the $CMR_{O2}$ PET map. Complexes I, III, and ATP synthase show no correlation with $CMR_{O2}$ (Complex I: rho = -0.09, $p_{spin,FDR}$ = 7.83e-01; Complex III: rho = 0.01, $p_{spin,FDR}$ = 9.74e-01; ATP synthase: rho = -0.09, $p_{spin,FDR}$ = 7.83e-01), consistent with the overall OXPHOS pattern.

Complex II shows a significant positive correlation with $CMR_{O2}$ (rho = 0.51, $p_{spin,FDR}$ = 4.99e-04). Complex IV, which directly utilizes oxygen as the terminal electron acceptor, shows only a moderate non-significant correlation (rho = 0.29, $p_{spin, FDR}$ = 3.87e-01).

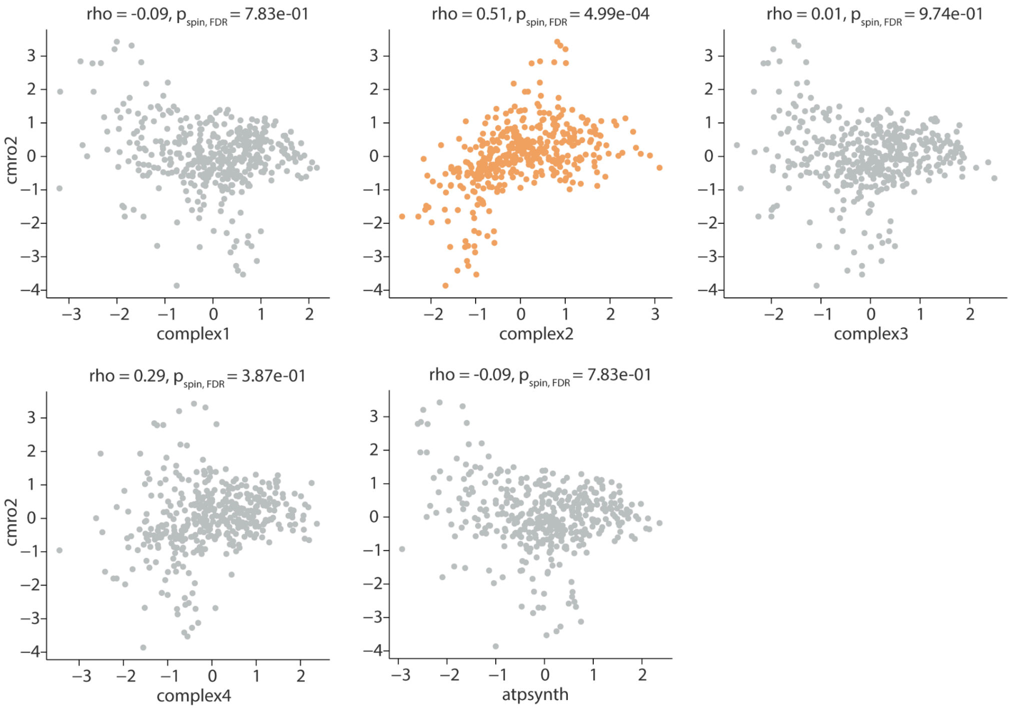


**- As I am not a neurologist or brain imaging specialist, I cannot comment on the choice of brain parcellation, functional network definitions, or cytoarchitecture interpretations.**

**Reviewer #2:**
**This study utilizes transcriptomic data from the Allen Human Brain Atlas (AHBA) alongside manually curated gene sets for metabolic pathways to construct and compare spatial cortical maps of five core energy metabolism pathways: glycolysis, the pentose phosphate pathway (PPP), the tricarboxylic acid (TCA) cycle, oxidative phosphorylation (OXPHOS), and lactate metabolism. These pathway maps are then aligned and analyzed in the context of multiscale structural and functional brain attributes, including network topology, cortical laminar architecture, cell-type specificity, etc. Here are my comments:**

**1. A recent study (https://can01.safelinks.protection.outlook.com/?url=https%3A%2F%2Fwww.nature.com%2Farticles%2Fs41586-025-08740-6&data=05%7C02%7Calain.dagher%40mcgill.ca%7Cfcc7eece6e1e4aabdca308ddea538bb3%7Ccd31967152e74a68afa9fcf8f89f09ea%7C0%7C0%7C638924366732991291%7CUnknown%7CTWFpbGZsb3d8eyJFbXB0eU1hcGkiOnRydWUsIlYiOiIwLjAuMDAwMCIsIlAiOiJXaW4zMiIsIkFOIjoiTWFpbCIsIldUIjoyfQ%3D%3D%7C0%7C%7C%7C&sdata=Rb7UXq8jUVpbGDTvpBmmIVDzB%2BRt9jWw2LnMQWvPt1U%3D&reserved=0) provides whole-brain spatial estimates of OXPHOS enzyme activity, mitochondrial DNA/volume density, and mitochondrial respiratory capacity via**

**multimodal MRI-based regression modeling. Please using this dataset to validate and interpret your metabolic pathway maps, especially for OXPHOS-related spatial patterns.**

We thank the reviewer for this suggestion. As noted in the Results and shown in Supplementary Figure S5, we have examined the spatial correspondence between our gene expression-based maps and the mitochondrial phenotype maps from Mosharov et al, 2025. We found that while TCA and OXPHOS maps do not align with mitochondrial density, they show significant correspondence with mitochondrial respiratory capacity.

> "Furthermore, comparison with interpolated *ex vivo* maps of mitochondrial activity profile [74] reveals spatial alignment with gene expression-based energy maps. While TCA and OXPHOS maps do not align with mitochondrial density, they correlate significantly with mitochondrial respiratory capacity, which reflects mitochondrial specialization across brain regions (Fig. S5)."

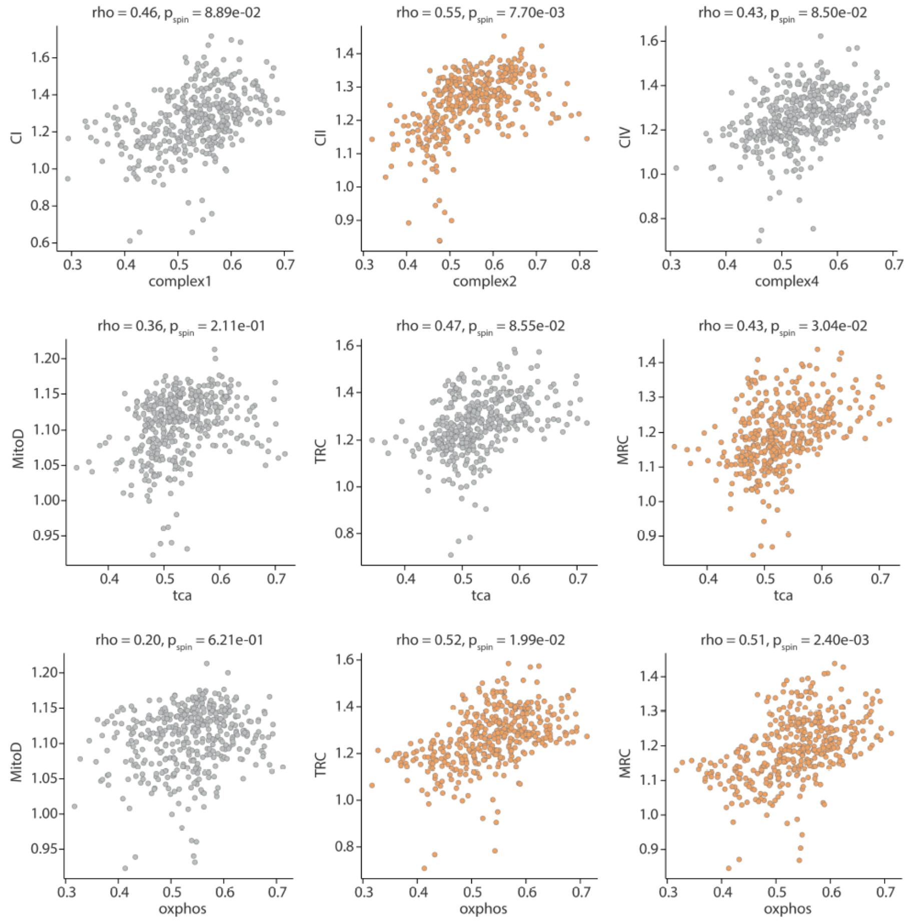


Figure S5. **Correlation between mitochondrial gene expression and phenotype maps.** Mitochondrial phenotype maps were obtained from Mosharov et al. [73] and parcellated into Schaefer-400. Orange scatter plots indicate statistically significant correlations (Spearman's) tested against 10 000 spatial-autocorrelation preserving nulls ($p_{spin} < 0.05$). The y-axis represents mitochondrial phenotype maps and the x-axis represents gene expression-based maps. CI, mitochondrial complex 1 activity; CII, mitochondrial complex 2 activity; CIV, mitochondrial complex 4 activity; MitoD, mitochondrial density; TRC, tissue respiratory capacity; MRC, mitochondrial respiratory capacity; tca, tricarboxylic acid cycle; oxphos, oxidative phosphorylation.

*References:*

1. *Mosharov, E. V. et al. A human brain map of mitochondrial respiratory capacity and diversity. Nature https://doi.org/10.1038/s41586-025-08740-6 (2025) doi:10.1038/s41586-025-08740-6.*

**2. Circadian gene expression is strongly influenced by energy constraints (PMID: 37692081). Please describe the spatial distribution and expression profiles of circadian regulators, such as BMAL1, CLOCK, PER, and CRY genes within your cortical pathway maps.**
**Their inclusion could offer mechanistic insight into the interplay between circadian regulation and brain energy metabolism.**

Circadian regulation and energy metabolism are indeed closely interconnected [1]. We have examined the spatial correspondence between core circadian clock genes (*ARNTL/BMAL1, ARNTL2/BMAL2, CLOCK, NPAS2*) and genes connected via transcriptional-translational feedback loops (core loop: *CRY1, PER1/2/3*, second loop: *NR1D1/2, RORA/B*, third loop: *DBP*) [2, 3] and our energy pathway maps.

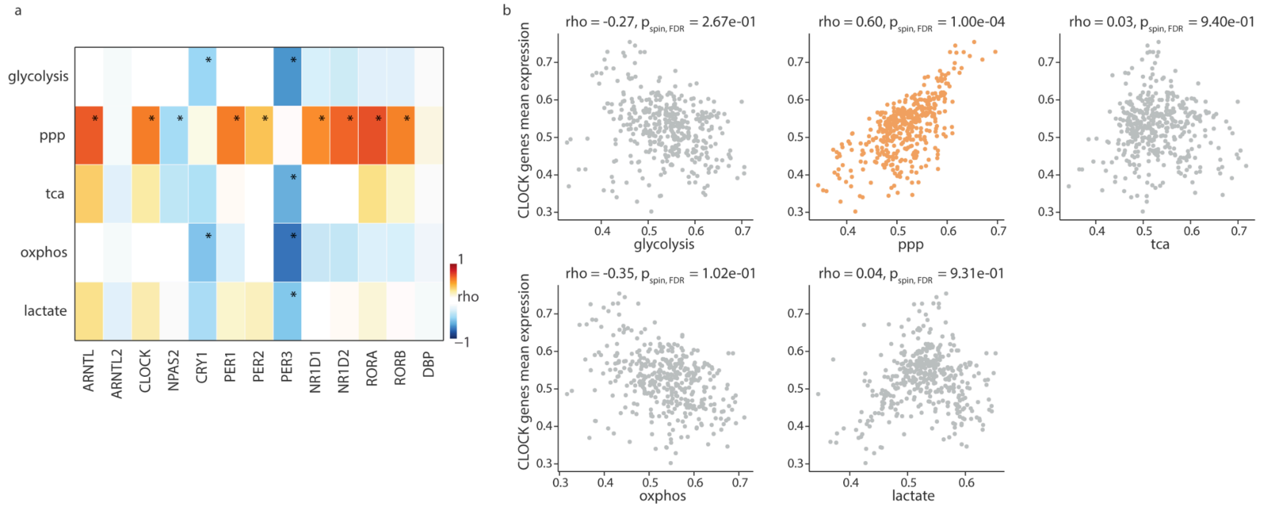


(panel a) Spatial alignment between energy pathway maps and core genes involved in the circadian rhythm in the Schaefer-400 parcellation. Colorbar represents Spearman's correlation value. Asterisks indicate statistical significance from spatial permutation testing with 10,000 nulls and FDR correction.
(panel b) Correlation between energy pathway map and average expression of all clock genes shown in panel a. Orange scatter plots indicate statistically significant correlations (Spearman's) tested against 10,000 spatial-autocorrelation preserving nulls after FDR correction ($p_{spin, FDR} < 0.05$).

As shown in the figure, PPP expression shows the strongest positive associations with several circadian genes, including *BMAL1, CLOCK, and PER1/2*. This observation is consistent with the PPP's role in generating molecular precursors for tissue homeostasis and DNA repair mechanisms [4].

However, a comprehensive investigation of circadian-metabolic interactions would require analysis of the full circadian machinery and downstream pathways, which are beyond the scope of the current study. We agree this represents an interesting direction for future research to elucidate how circadian regulation relates to the spatial organization of brain energy metabolism.

*References:*


*1. Cox, K. H. & Takahashi, J. S. Circadian clock genes and the transcriptional architecture of the clock mechanism. J. Mol. Endocrinol. 63, R93–R102 (2019).*

*2. Dong, Y., Lam, S. M., Li, Y., Li, M.-D. & Shui, G. The circadian clock at the intersection of metabolism and aging – emerging roles of metabolites. J. Genet. Genomics S1673852725001237 (2025).*

*3. Sancar, G. & Brunner, M. Circadian clocks and energy metabolism. Cell. Mol. Life Sci. 71, 2667–2680 (2014).*

*4. Rey, G. et al. The Pentose Phosphate Pathway Regulates the Circadian Clock. Cell Metab. 24, 462–473 (2016).*


**3. The final PPP gene set listed in the manuscript ("Energy metabolic pathway gene sets" table) includes only five genes (PGD, PRPS2, RBKS, RPEL1, and RPIA), omitting several well-established key PPP enzymes. Please clarify the rationale for excluding other canonical genes (e.g., G6PD) and discuss how this may impact the completeness or interpretability of the PPP spatial map.**

The final gene sets used to create the pathway expression maps have fewer members than the curated gene sets retrieved from Gene Ontology and Reactome databases. This is because some genes are not present in the Allen Human Brain Atlas, while some other genes do not pass our quality control criterion of differential stability (ds > 0.1).

Retrieved gene sets for the PPP from GO and Reactome databases had 21 and 15 members, respectively, out of which 12 genes were annotated in both databases. Among the 12 genes, seven genes did not meet the differential stability threshold, including key oxidative branch enzymes *G6PD* and *PGLS* and therefore had to be excluded from the pathway gene expression matrix.

We mention this point in the beginning of the results section that the PPP map mainly includes genes from the non-oxidative branch and show how this might affect the weak alignment observed between the ppp and the glycolysis and OXPHOS expression maps (Supplementary Figure S1):

> "Conversely, given the PPP's function in cellular defense against ROS, it could be anticipated that regions with high oxidative metabolism would exhibit relatively elevated PPP activity. However, the PPP gene set used here predominantly includes genes involved in its anabolic nonoxidative branch (i.e. *PRPS2, RBKS, RPEL1, RPIA*), responsible for the production of 5-carbon sugars [6, 47]. Indeed, when we look into the *PGD* gene, the only gene representing the oxidative branch in our PPP gene set, which also directly catalyzes one of the NADPH-producing reactions, we find significant spatial correlations with glycolysis and OXPHOS maps (glycolysis: rho = 0.75, $p_{spin}$ = 1×10−4; OXPHOS: rho = 0.71, $p_{spin}$ = 1×10−4; Fig. S1)."

Furthermore, in our interpretation of the PPP findings, we have mostly focused on cellular repair processes relevant to the non-oxidative branch rather than the antioxidant functions primarily associated with the oxidative branch. For example, in the results and discussion we mention:

> "There is a moderate alignment between $CMR_{O2}$ and the PPP map (rho = 0.54, $p_{spin,FDR}$ = 0.002), potentially highlighting greater need for cellular repair processes in regions with greater oxygen consumption and consequently higher oxidative damage."

> "The higher expression of PPP in primary sensory cortices may potentially reflect the greater demand for robust sensory encoding [53], necessitating active maintenance of synaptic integrity through ongoing cellular biosynthesis."

We have also revised the one place where we interpreted PPP's role in antioxidant defense in the discussion:

> "This alignment may reflect the recruitment of both energy-producing and anabolic pathways to support the metabolic and antioxidant demands of these cells."

To:

> "This alignment likely reflects the recruitment of both energy-producing and anabolic pathways to support the metabolic and cellular repair processes in these cells"

Finally, these two key PPP genes (*G6PD* and *PGLS)* are included in our sensitivity analysis where we created the gene expression maps without any differential stability threshold and we show that the spatial distribution remains consistent with our original map. The gene sets for this sensitivity analysis have been added in Supplementary Table S6.

| Pathway | Genes |
|---|---|
| Glycolysis | *ADPGK, ALDOA, ALDOC, BPGM, ENO1, ENO2, ENO3, ENO4, GAPDH, GAPDHS, GCK, GPI, PFKFB2, PFKL, PFKM, PFKP, PGAM1, PGAM2, PGK1, PPP2CA, PRKACA, TPI1* |
| PPP | *DERA, G6PD, PGD, PGLS, PRPS2, RBKS, RPE, RPEL1, RPIA, SHPK, TALDO1, TKT* |
| TCA | *ACO2, CS, DLST, FH, IDH2, IDH3A, IDH3B, IDH3G, MDH2, NNT, OGDH, SDHA, SDHB, SDHC, SDHD, SUCLA2, SUCLG1, SUCLG2* |
| OXPHOS | *ATP5F1A, ATP5F1B, ATP5F1C, ATP5F1D, ATP5ME, ATP5MF, ATP5MG, ATP5PB, ATP5PD, ATP5PF, ATP5PO, COX4I1, COX5A, COX5B, COX6A1, COX6B1, COX6C, COX7A2L, COX7B, COX7C, COX8A, CYC1, CYCS, NDUFA1, NDUFA10, NDUFA2, NDUFA3, NDUFA4, NDUFA5, NDUFA6, NDUFA7, NDUFA8, NDUFA9, NDUFAB1, NDUFAF1, NDUFB1, NDUFB10, NDUFB2, NDUFB3, NDUFB4, NDUFB5, NDUFB6, NDUFB7, NDUFB8, NDUFB9, NDUFC1, NDUFC2, NDUFS1, NDUFS2, NDUFS3, NDUFS4, NDUFS5, NDUFS6, NDUFS7, NDUFS8, NDUFV1, NDUFV2, NDUFV3, SDHA, SDHB, SDHC, SDHD, UQCR10, UQCR11, UQCRB, UQCRC1, UQCRC2, UQCRFS1, UQCRH, UQCRQ* |
| Lactate | *ACACB, EMB, GATD1, HAGH, HAGHL, HIF1A, LDHA, LDHAL6A, LDHB, LDHC, LDHD, MRS2, PARK7, PER2, PFKFB2, PNKD, SLC16A1, SLC16A3, SLC16A7, SLC16A8, SLC37A4, SLC5A12, TIGAR* |

“TABLE S6. Energy metabolic pathway gene sets without the differential stability threshold. As before, gene sets were produced based on GO biological processes and Reactome pathway IDs. Genes annotated in both databases used for the analyses are listed. PPP, pentose

**Reviewer #3: Pourmajidian and collaborators present an integrative analysis combining energy metabolism pathways, transcriptomics, and multi-scale structural and functional data of the human brain. Their study provides an interesting example of how different kinds of available data resources can be reused and combined to explore an interesting biological problem: the distribution of energy metabolism pathway potential across the human brain. Among their main findings, they report an heterogeneous spatial distribution of energy metabolism pathways across structural and functional areas as estimated by gene expression, a dichotomy in these patterns between ATP production and anabolic pathways, and (un)expected correlations between pathway gene expression and neurophysiological activity measurements. More broadly, they propose that gene expression can be used to study the metabolic makeup of the human brain across space and developmental time.**

**The motivation, analyses, and results are presented clearly. I have some comments intended to improve some aspects of the analysis and interpretation.**

**The authors use multiple neurophysiological datasets to correlate with gene expression. Unlike standard neurophysiological measurements, there is no such thing as a standard cell type composition map. Rather, cell type composition is also inferred from the bulk transcriptomic data they use to estimate metabolic pathway potential. A brief explanation**

**of this contrasting approach and the underlying assumptions would improve the results section presenting this analysis -- which is in between all other standard methods.**

We have now clarified this methodological detail in the results section “Energy correlates of multi-scale cortical features” that distinguishes the cell type composition and laminar organization maps from the direct physiological measurements (PET and MEG):

> “It should be noted that, unlike the PET and MEG maps which represent direct physiological measurements, cell type composition and laminar organization maps are derived from the AHBA using cell- and layer-specific gene markers as proxies for cell-type abundance and cortical laminar structure [67, 69, 70]”

**Related to the previous point, given the nonstandard measurement, a presentation of cell type estimations across brain regions, how variable and correlated they are, and how these measurements fit or not with expectations based on literature would strengthen the analyses. The authors may consider using similar individual markers to contrast with their estimates as those used in the reference they cite (Wagstyl et al. 66).**

We have performed additional analyses to characterize cell type distributions across the cortex:

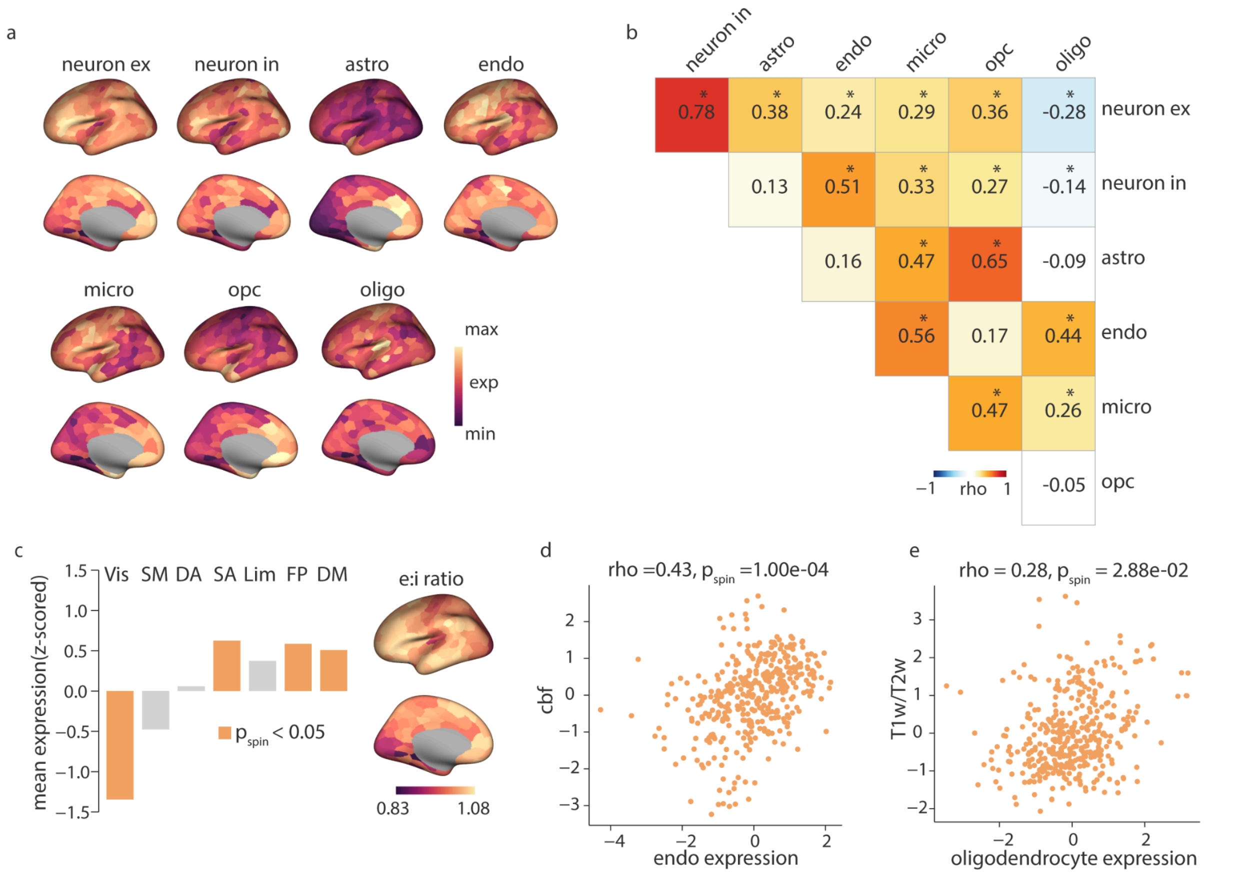

(panel a) The spatial distribution of cell type marker expression across the 400 cortical regions in the Schaefer parcellation.
(panel b) pairwise correlations between cell-type maps shown in panel a. Asterisks indicate statistical significance tested against 10,000 spatial nulls and after FDR correction. Color bar shows the strength of the pair-wise correlation (Spearman's rho).
(panel c) Distribution of the E:I ratio across the seven resting state functional networks.
(panel d) Spatial alignment between endothelial gene expression and cerebral blood flow from PET imaging.
(panel e) Spatial alignment between Oligodendrocyte gene expression and T1w/T2w cortical map. Dots in the scatterplot represent the 400 cortical regions in the Schaefer parcellation.

As shown in the figure, excitatory and inhibitory neurons show a strong positive correlation (rho = 0.78, $p_{spin, FDR}$ = 0.0002), consistent with their functional coupling in neural circuits. Moreover, excitatory neurons correlate moderately with astrocytes (rho = 0.38, $p_{spin,FDR}$ = 0.002), reflecting their metabolic interdependence [1]; and endothelial cells correlate with microglia (rho = 0.56, $p_{spin, FDR}$ = 0.0002), in line with their coordinated roles in neurovascular coupling [2, 3].

Due to the absence of cellular composition maps, direct validation of these estimates is challenging. We attempted three analyses: First, we examined the excitatory-to-inhibitory neuron (E:I) ratio, which has been reported to follow the sensory-association axis. Our gene expression-based e:i ratio shows lower values in visual and primary sensorimotor cortices, and greater values in higher order association regions including frontoparietal and default mode networks (panel c), consistent with previous reports [4, 5]. Second, endothelial cell marker shows a moderate correlation with PET-measured cerebral blood flow (rho = 0.43, $p_{spin}$ = $1\times10^{-4}$; panel d), providing some validation of these cell type estimates. Third, the oligodendrocyte expression map shows a significant positive correlation with the intra-cortical myelin from T1w/T2w map (rho = 0.28, $p_{spin}$ = 2.88e-02 , panel e).

Overall, in the absence of direct measures, cell-type marker gene expression can provide a proxy to study cell-type abundance across the cortical regions [6, 7].

*References:*


*1. Magistretti, P. J. & Allaman, I. Lactate in the brain: From metabolic end-product to signalling molecule. Nature Reviews Neuroscience vol. 19 235–249 (2018).*

*2. Császár, E. et al. Microglia modulate blood flow, neurovascular coupling, and hypoperfusion via purinergic actions. J. Exp. Med. 219, e20211071 (2022).*

*3. Kisler, K., Nikolakopoulou, A. M. & Zlokovic, B. V. Microglia have a grip on brain microvasculature. Nat. Commun. 12, 5290 (2021).*

*4. Sydnor, V. J. et al. Neurodevelopment of the association cortices: Patterns, mechanisms, and implications for psychopathology. Neuron vol. 109 2820–2846 (2021).*

5. Goulas, A. et al. The natural axis of transmitter receptor distribution in the human cerebral cortex. https://doi.org/10.1073/pnas.2020574118/-/DCSupplemental.y doi:10.1073/pnas.2020574118/-/DCSupplemental.y.

6. Seidlitz, J. et al. Transcriptomic and cellular decoding of regional brain vulnerability to neurogenetic disorders. Nat. Commun. 11, 3358 (2020).

7. Burt, J. B. et al. Hierarchy of transcriptomic specialization across human cortex captured by structural neuroimaging topography. Nat. Neurosci. 21, 1251–1259 (2018).

**The authors find significant positive correlations between inhibitory neurons and all energy metabolism maps. They attribute this association to their fast-spiking activity. However, not all inhibitory neurons are fast-spiking, thus considering only one class likely confounds composition estimates. Based on data on Wagstyl et al. 66, it seems clear that the distribution of markers of specific excitatory and inhibitory neuron subtypes is heterogeneous. This provides an opportunity to more directly contrast to what extent average expression of metabolic pathways correlates with expression of fast-spiking (e.g., PV-expression) neurons and not others. This kind of more targeted analysis would be a nice addition to the study to further support their interpretation/explanation.**

We thank the reviewer for this helpful suggestion. We agree that the inhibitory neuron population is heterogeneous, and the observed correlations may be driven by specific subtypes. To test whether fast-spiking neurons underlie these associations, we examined correlations between energy pathway maps and individual inhibitory markers including pan-GABAergic neurons (*GAD1, GAD2*), parvalbumin (*PVALB*), somatostatin (*SST*), calbindin (*CALB*), and *VIP*.

Parvalbumin expression, the canonical marker of fast-spiking interneurons, shows significant positive correlations with the PPP (rho = 0.72, $p_{spin}$ = 0.004), TCA (rho = 0.62, $p_{spin}$ = 0.043) and lactate (rho = 0.65, $p_{spin}$ = 0.009) maps. Correlations with glycolysis (rho = 0.24, $p_{spin}$ = 0.71) and OXPHOS (rho = 0.28, $p_{spin}$ = 0.65) do not reach statistical significance in contrast with our original analysis. Other inhibitory subtypes show weaker or negative correlations, suggesting that the metabolic signature we observed does indeed reflect the specific bioenergetic demands of fast-spiking parvalbumin-positive neurons rather than inhibitory neurons as a whole. This interpretation aligns with reports of greater metabolic demand and oxidative stress damage in fast-spiking cells [1], though future high-resolution cell-specific datasets would be needed to fully and robustly characterize these relationships.

We have added a statement regarding these finding in the results section “Energy correlates of multi-scale cortical features”:

"To test whether these associations are attributable to the fast-spiking population, we further used individual inhibitory neuron marker genes. Parvalbumin expression shows a significant correlation with the PPP, TCA and lactate maps (Fig. S6), suggesting that the metabolic signature we observe may reflect the unique energy demands of parvalbumin-positive neurons."

And revised the corresponding paragraph in the discussion based on these new analyses:

"We also find that inhibitory neurons show spatial overlap across all energy pathways. Specifically, parvalbumin expression, the canonical marker of fast-spiking interneurons, shows pronounced alignment with the PPP, TCA and lactate maps (Fig. S6). The high-frequency spiking activity of parvalbumin-positive neurons imposes an energetic load on the brain, evidenced by the abundance of mitochondria in these cells [112–115], which renders this cell population especially vulnerable to oxidative damage [116, 117]; This alignment likely reflects the recruitment of both energy-producing and anabolic pathways to support the metabolic and cellular repair processes in these cells."

We also produced cortical maps for layer-specific excitatory neurons using individual marker genes from the literature [2, 3, 4]. Consistent with our original interpretation, markers for Layer 5 excitatory neurons and Betz cells show the strongest correlations with ATP-producing pathways (glycolysis, OXPHOS), hinting at the high energy demands of these long-projection neurons.

Results for non-neuronal cells remain generally consistent when using individual marker genes.

We have included the inhibitory and excitatory neuronal markers in the supplementary materials (Supplementary Figure S6 and Supplementary Table S3).

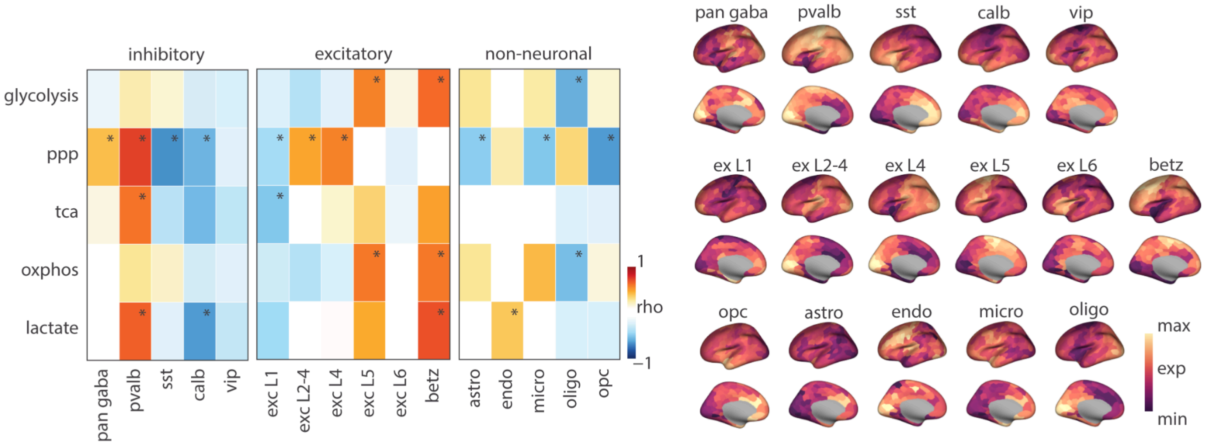


"Figure S6. Spatial alignment between energy maps and individual inhibitory and excitatory marker genes. For each individual sub-type, gene markers were obtained from Kang et al. [91] and Hodge et al. [180]. For the list of gene markers see Table S3. (Left) Heatmap showing the

strength of correlation between energy and cell-type maps. Colorbar indicates Spearman's correlation values. Asterisks indicate statistical significance when tested against a distribution of 10 000 spatial-autocorrelation preserving nulls after FDR-correction for multiple comparisons. (Right) Cortical distribution of neuronal sub-types gene expression. Colorbar shows expression values across the 400 Schaefer regions. Cell types: pvalb, parvalbumin; sst, somatostatin; calb, calbindin; vip, vasoactive intestinal peptide."

Cell type marker genes used to create these additional maps are now added in Supplementary Table S3.

TABLE S3. **Cell-type marker genes.** Individual cell-type markers used to produce cortical maps for inhibitory and excitatory neuronal subtypes. Marker genes were obtained from Kang et al. [92], Hodge et al. [183].

| Cell type | Marker genes |
|---|---|
| Pan GABAergic | *GAD1, GAD2* |
| Parvalbumin+ | *PVALB* |
| Somatostatin+ | *SST* |
| Calbindin+ | *CALB1, CALB2* |
| VIP+ | *VIP* |
| Layer 1 Excitatory | *RELN* |
| Layer 2-4 Excitatory | *CUX1, UNC5D* |
| Layer 4 Excitatory | *RORB* |
| Layer 5 Excitatory | *BCL11B, ETV1, FEZF2* |
| Layer 6 Excitatory | *FOXP2, NTSR1, SOX5, SSTR2, TBR1, TLE4, ZFPM2* |
| Betz Cell | *ASGR2* |
| Astrocyte | *AQP4, GFAP, S100B* |
| Endothelial Cell | *CLDN5* |
| Microglia | *TYROBP* |
| Oligodendrocyte | *MBP* |
| Oligodendrocyte Precusor Cell | *CSPG4, PDGFRA* |

"TABLE S3. Cell-type marker genes. Individual cell-type markers used to produce cortical maps for inhibitory and excitatory neuronal subtypes. Marker genes were obtained from Kang et al. [91], Hodge et al. [180]."

These results are also added in the results section about the energy profile of cortical laminar structure:

> "The glycolysis and the OXPHOS maps show significant alignment with cortical layer 5 (glycolysis: rho = 0.73, $p_{spin,FDR}$ = 0.001, OXPHOS: rho = 0.69, $p_{spin,FDR}$ = 0.001). Specifically, individual markers of excitatory layer 5 and Betz cells show significant correlations with glycolysis and OXPHOS (Fig. S6). This could hint to the greater metabolic demand of large pyramidal cells with their extensive subcortical projections [83, 84]."

We have also added a reference to this new supplementary figure in the discussion:

> “Conversely, glycolysis and OXPHOS spatially align with the infragranular layer 5, the main output layer of the cortex, reflecting the greater static energy requirements of large pyramidal neurons such as Betz cells (Fig. S6).”

*References:*


*1. Galow, L. V. et al. Energy substrates that fuel fast neuronal network oscillations. Front. Neurosci. 8, (2014).*

*2. Kang, H. J. et al. Spatio-temporal transcriptome of the human brain. Nature 478, 483–489 (2011).*

*3. Hodge, R. D. et al. Conserved cell types with divergent features in human versus mouse cortex. Nature 573, 61–68 (2019).*

*4. Wagstyl, K. et al. Transcriptional cartography integrates multiscale biology of the human cortex. eLife 12, RP86933 (2024).*


**The authors analyse the correlation of individual genes within energy pathways and CMRglc and notice the high variability. This suggests that a single average value might not necessarily be a good representation of metabolic potential, but might be biased to specific genes. An additional sensitivity test to address this point would strengthen the analytical choice. The PCA analysis in Fig. S2 relates to this. How consistent are PCs vs average? In a given comparison with a spatial map, how variable are the correlation estimates of individual genes and how much they deviate from the correlation of the average expression. I appreciate that some of these limitations are mentioned in the discussion section.**

We agree that the average expression is a simplified measure and may not fully capture the heterogeneity among genes within each metabolic pathway, as we mention in the limitations of our study in the discussion section:

> “Second, energy metabolism is characterized by interconnected pathways that branch out and converge via shared enzymes and metabolites [9]. Studying these pathways in isolation and relying on average expression is an oversimplification of their complex dynamics.”

Nonetheless, our choice to use the average expression was motivated by interpretability, as it provides a direct measure of regional gene expression, assigning equal weight to each pathway member, whereas PCA emphasizes dominant variance patterns that may be disproportionately driven by a subset of genes.

To evaluate the robustness of this choice and as suggested by the reviewer, we repeated our analyses using the first principal component (PC1) of pathway gene expression as an alternative summary measure.

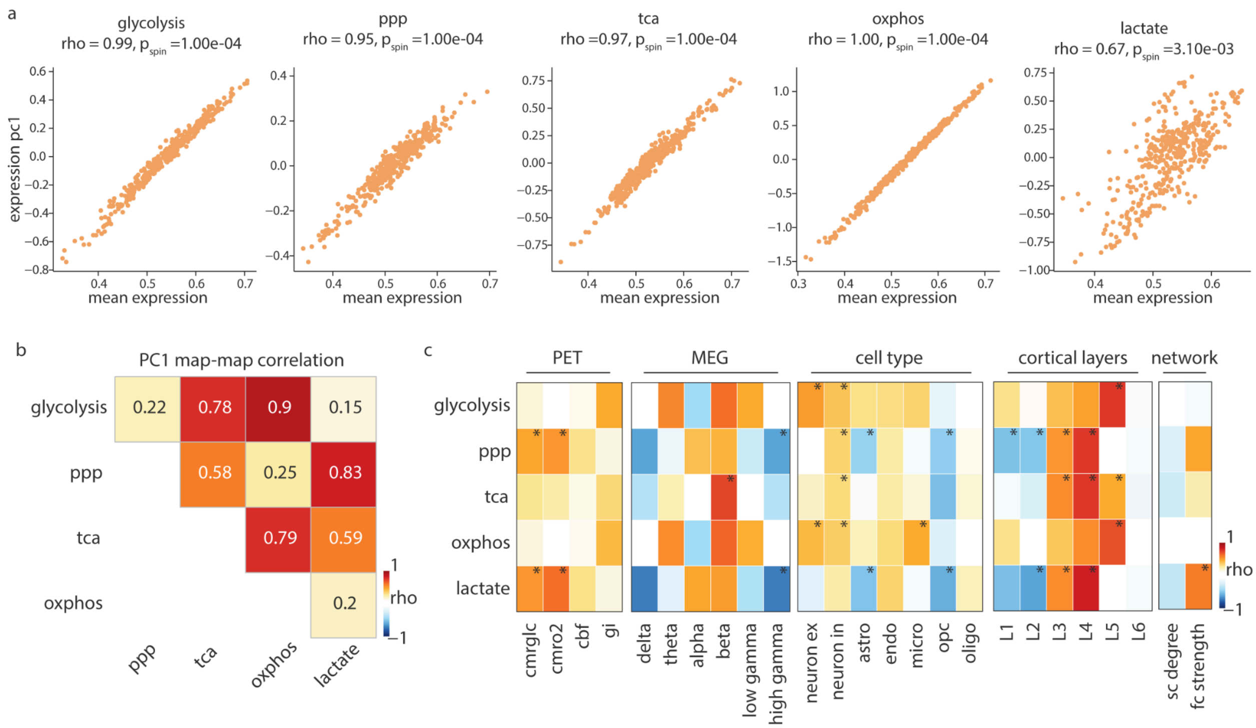


"Figure S11. Energy maps produced from the first principal component of pathway gene expression. (a) Alignment between the mean gene expression maps and the first principal component of gene expression for each energy pathway. Dots in the scatterplot represent 400 cortical regions in the Schaefer parcellation. (b) Spatial alignment among energy PC1 maps calculated using pairwise Spearman's correlation. (c) Pairwise Spearman's correlation of PC1 energy maps and multi-scale cortical features. Asterisk represents significance after permutation testing using 10 000 spatial nulls and FDR correction for multiple comparison. Colorbar shows Spearman's correlation values."

As shown in panel (a), the mean and PC1 maps are highly correlated across all pathways (glycolysis: rho = 0.99, PPP: rho = 0.95, TCA: rho = 0.97, OXPHOS: rho = 1.00, lactate: rho = 0.67; all $p_{spin} < 0.01$), indicating that both approaches capture consistent spatial gradients. The lactate map shows a lower correspondence between PC1 and mean expression compared to other metabolic pathways; unlike the other gene sets which correspond to discrete biochemical pathways (glycolysis, TCA cycle, OXPHOS, PPP), the lactate gene set encompasses a collection of lactate-related processes including production, consumption, and transport of lactate. This more ambiguous "pathway" definition for lactate may underlie the deviation we observe between PC1 and mean expression.

The overall strong convergence between PC1 and mean expression perhaps demonstrates the presence of a pathway-level spatial pattern that is not dependent on the specific aggregation method. Furthermore, we show that the alignment among energy maps and between energy maps and multi-scale cortical features remain overall consistent when using the PC1 maps, with the exception of the lactate map (panels b and c).

We have added this analysis in the sensitivity section of the results:

> "Fourth, to evaluate the robustness of our results to the choice of summary measure, we repeated the analysis using the first principal component (PC1) of gene expression for each pathway. The mean and PC1 maps are highly correlated and show consistent alignment with multi-scale cortical features (Fig. S11)."

Regarding the variability in gene-wise correlations as we have shown for $CMR_{Glc}$ in Figure 5: We further examined the gene-wise correlations with regards to other maps including: $CMR_{O2}$, MEG beta oscillations, excitatory and inhibitory neurons, and cortical layers IV and V.

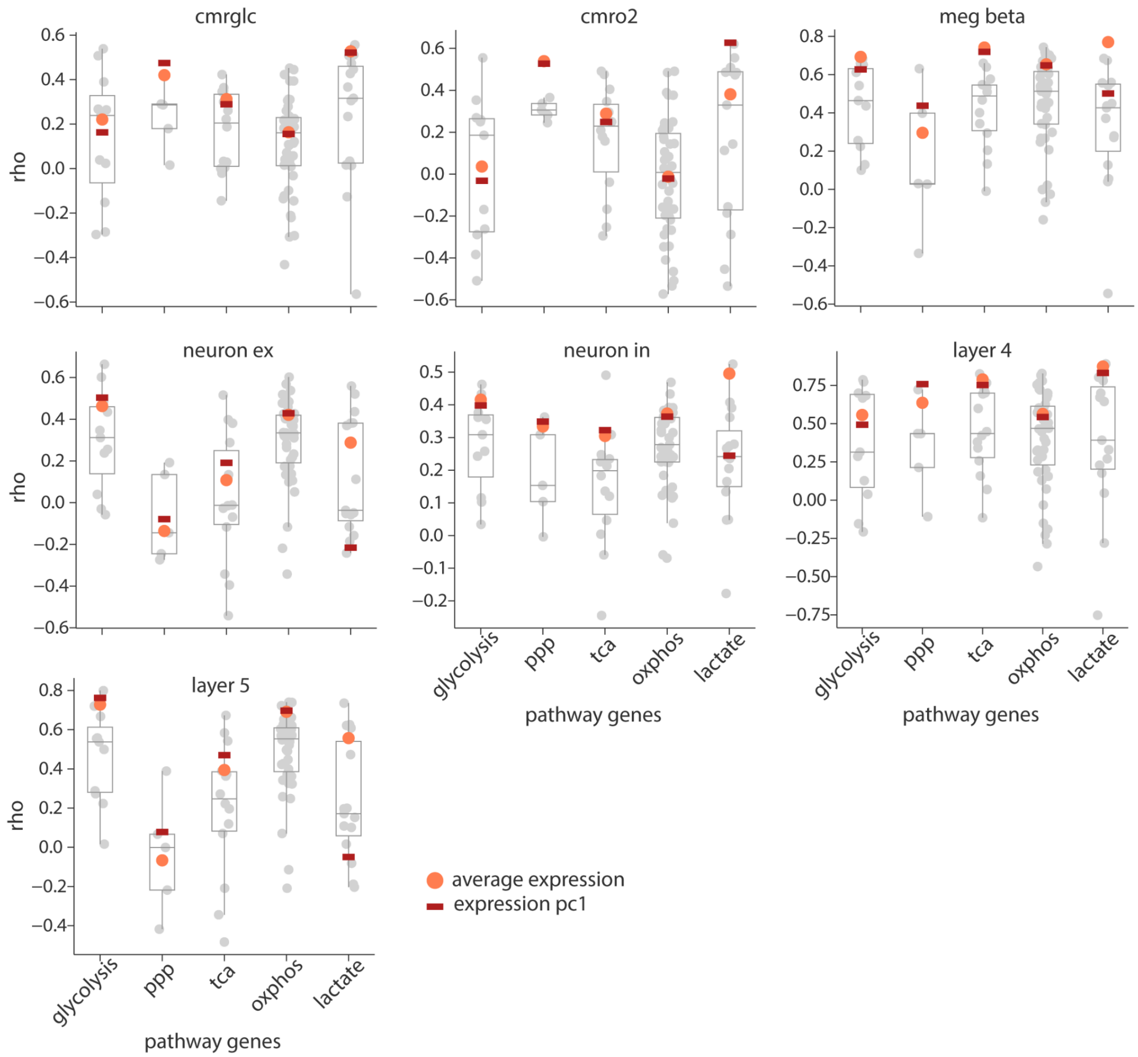

As shown in the figure, individual genes within each pathway exhibit substantial variability in their correlations with other spatial maps, potentially explained by transcriptional, post-translational modifications and regulatory mechanisms or varying biological roles within and beyond the core metabolic pathway. Despite this variability, the mean expression and PC1 correlations remain highly consistent with each other, suggesting that both summary measures capture a shared pathway-level spatial signal above that of individual gene heterogeneity. Importantly, the fact that pathway-level summaries (mean/PC1) often show stronger correlations than individual genes perhaps occurs because averaging reduces gene-specific measurement noise, while amplifying the shared pathway-level spatial pattern driven by regional energetic demands.

**The authors do not observe a significant correlation between glycolysis/OXPHOS maps and metabolic PET imaging estimates of resting-state glucose and oxygen consumption or cerebral blood flow. They interpret discrepancy as reflecting the fact that "PET measures energy usage at rest while gene expression is a measure of potential energy demand and supply during neuronal activation". Gene expression changes as captured with the resolution of bulk microarray snapshots likely reflect cellular variability and not changes in neuronal activity. Many of the correlations observed are likely explained by the differential expression of enzymes and metabolic pathway genes between the different cell types and subtypes with variable composition across the brain. This observation similarly applies to the developmental changes -- as consistent with the neuronal progenitor vs synapse analysis in Fig. S6. Discussing this additional interpretation in the discussion section would improve the results and interpretation.**

We apologize for the ambiguous explanation. By “potential energy demand and supply during neuronal activity”, we meant that gene expression likely reflects metabolic potential (i.e. respiratory capacity [1]) rather than real-time oxygen consumption at rest, as another reviewer pointed out. Additionally, mitochondrial electron transport chain proteins are among the most long-lived proteins in the cell [2, 3], potentially decoupling transcript levels from the oxygen consumption measured by PET.
We have now revised this sentence and clarified our interpretation in the discussion:

> “The weak correspondence between OXPHOS gene expression and $CMR_{O2}$ likely reflects several factors. Mitochondrial proteins are among the most long-lived within the cell, allowing sustained function even after transcript depletion [101, 102]. As a result, OXPHOS gene expression may better capture long-term metabolic capacity rather than acute oxygen use. In addition, $CMR_{O2}$ PET reflects resting oxygen consumption and does not account for spare respiratory capacity, which may be better indexed by transcriptional profiles.”

*References:*

*1. Marchetti, P., Fovez, Q., Germain, N., Khamari, R. & Kluza, J. Mitochondrial spare respiratory capacity: Mechanisms, regulation, and significance in non-transformed and cancer cells. FASEB J. 34, 13106–13124 (2020).*

*2. Bomba-Warczak, E. & Savas, J. N. Long-lived mitochondrial proteins and why they exist. Trends Cell Biol. 32, 646–654 (2022).*

*3. Krishna, S. et al. Identification of long-lived proteins in the mitochondria reveals increased stability of the electron transport chain. Dev. Cell 56, 2952-2965.e9 (2021).*

We agree that the regional heterogeneity in energy pathway expression also reflects underlying microstructural differences, including cellular composition and laminar organization. We demonstrate this extensively through the alignment of metabolic maps with multi-scale cortical features in Figure 5 and state this in the results section “energy correlates of multi-scale cortical features”:

> “The heterogeneity of energy pathway profiles likely arises from the distinct molecular and cellular properties of different cortical regions at the microscale, as well as neurophysiological and network-level attributes at the macroscale. Cortical regions exhibit variability in their glucose uptake and oxygen consumption, as shown by molecular imaging. Neurophysiological activity associated with signaling (i.e., action potentials and synaptic transmission) are energy-intensive and account for the majority of cortical energy demand [1, 5, 8, 65]. Cellular specialization further shapes energy metabolism, as glial and neuronal populations exhibit distinct metabolic profiles [9].”

**Minor technical comment:**
**The authors may consider including as negative control the expression of genes not expected to vary across brain regions to support the specificity of metabolic pathway changes -- e.g., any housekeeping gene(s).**

Identifying appropriate housekeeping genes as controls is challenging in the context of whole-brain regional comparisons. Unlike controlled homogenous cell culture systems with uniform density, the brain shows substantial regional variation in cellular density and cell-type composition, as the reviewer has mentioned. Even canonical housekeeping genes, typically used to normalize expression in homogeneous samples, vary across brain regions due to these structural differences. For example, *GAPDH*, one of the most commonly used housekeeping genes, is itself a glycolytic enzyme and shows regional variation. Similarly, cytoskeletal genes often used as controls (e.g., *ACTB*) would vary with neuronal density and morphology across regions.

**Additional changes:**

1. We have now added branched chain amino acid (BCAA) catabolism pathway to the set of extended maps. BCAA catabolism is closely linked to energy metabolism and we have therefore added both the BCAA catabolism cortical map in Supplementary Figure S16 and the lifespan trajectory of genes in this pathway in Supplementary Figure S19.

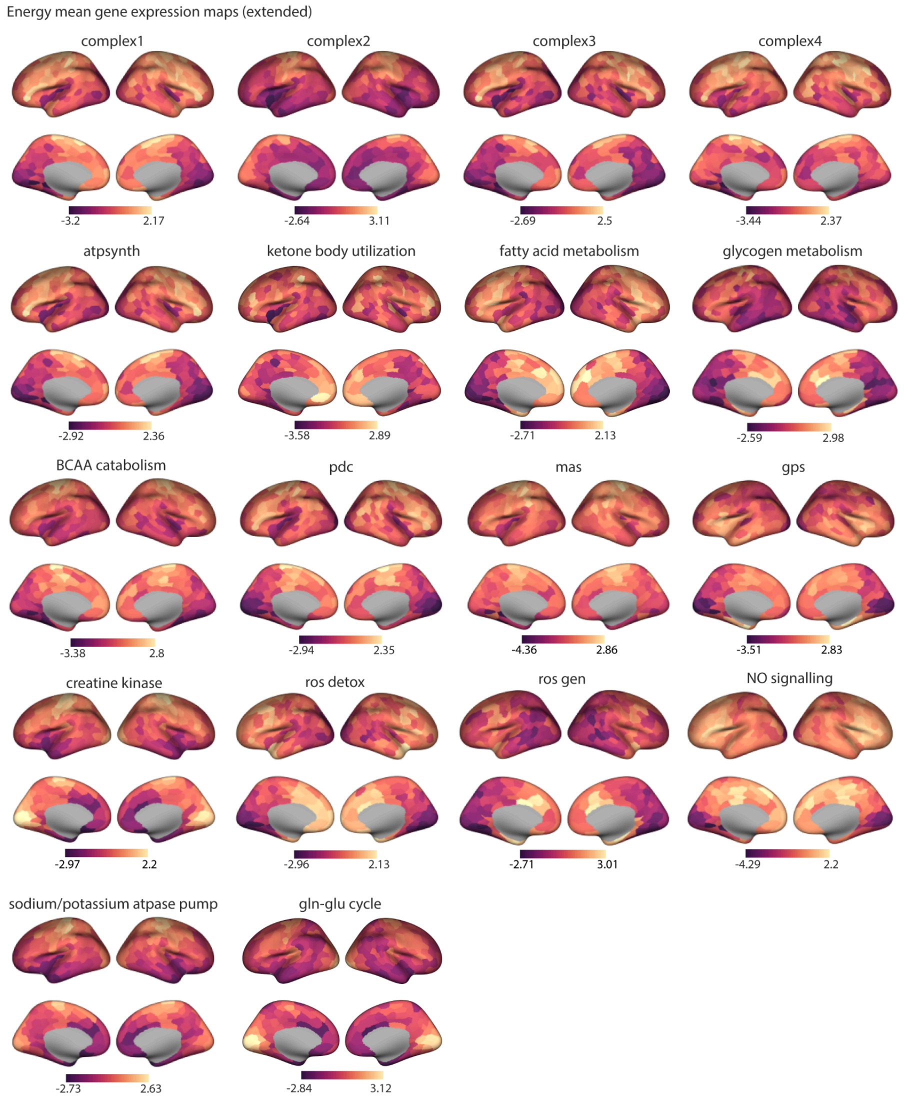


"Figure S16. Extended set of energy related pathways. Mean gene expression maps were produced as before (see Methods). Color bars represent mean gene expression, z-scored across the 400 cortical regions in the Schaefer-400 parcellation. The clustering analysis of these maps can be found in Fig. S17. For completeness, the lifespan trajectory of the extended set of energy maps can be found in Fig. S19. atpsynth, ATP synthase complex; BCAA, branch chained amino acid; pdc, pyruvate dehydrogenase complex; mas, malate-aspartate shuttle; gps: glycerol-3-phosphate shuttle; ros detox, detoxification of reactive oxygen species; ros gen, generation of reactive oxygen species; no signaling, nitric oxide signaling; gln-glu cycle, glutamine-glutamate cycle"

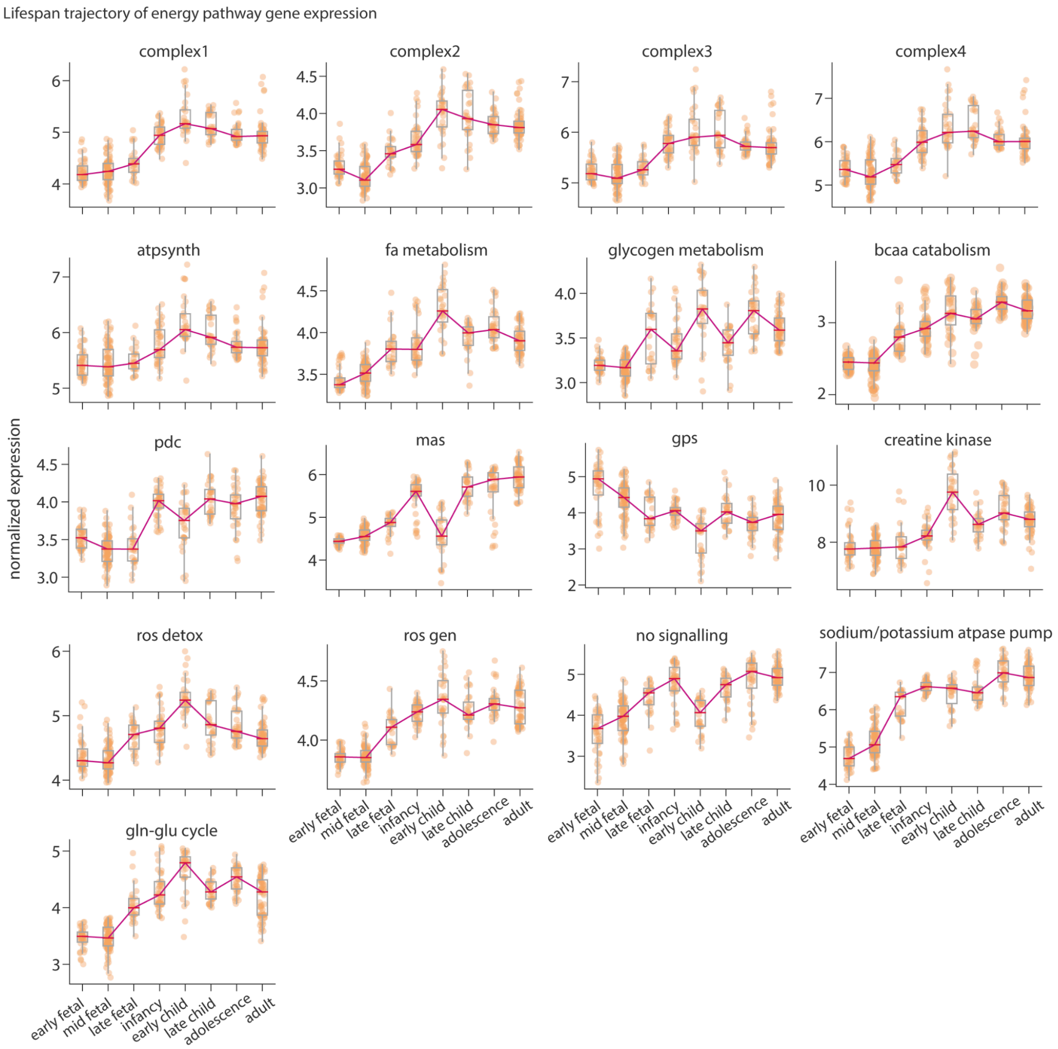


“Figure S19. Lifespan trajectory of the extended energy metabolism maps. Developmental trajectories of energy metabolism pathways were produced using the BrainSpan RNA-seq expression dataset. For each energy pathway, mean expression was calculated across all genes for each sample and median expression across all samples falling into each age group was calculated. Analysis only included cortical regions. The y-axis represents normalized log2(RPKM) expression values. Dots represent individual samples in each age group. Line plot depicts the trajectory of median gene expression. atpsynth, ATP synthase complex; pdc, pyruvate dehydrogenase complex; mas, malate-aspartate shuttle; gps: glycerol-3-phosphate shuttle; ros detox: detoxification of reactive oxygen species; ros gen, generation of reactive oxygen species; no signalling, nitric oxide signaling; gln-glu cycle, glutamine-glutamate cycle.”

2. We have now changed the supplementary figure regarding the clustering of extended energy maps from Louvain’s modularity-maximization clustering to hierarchical clustering. This new clustering figure is more informative as to the relationship between the extended energy pathways.

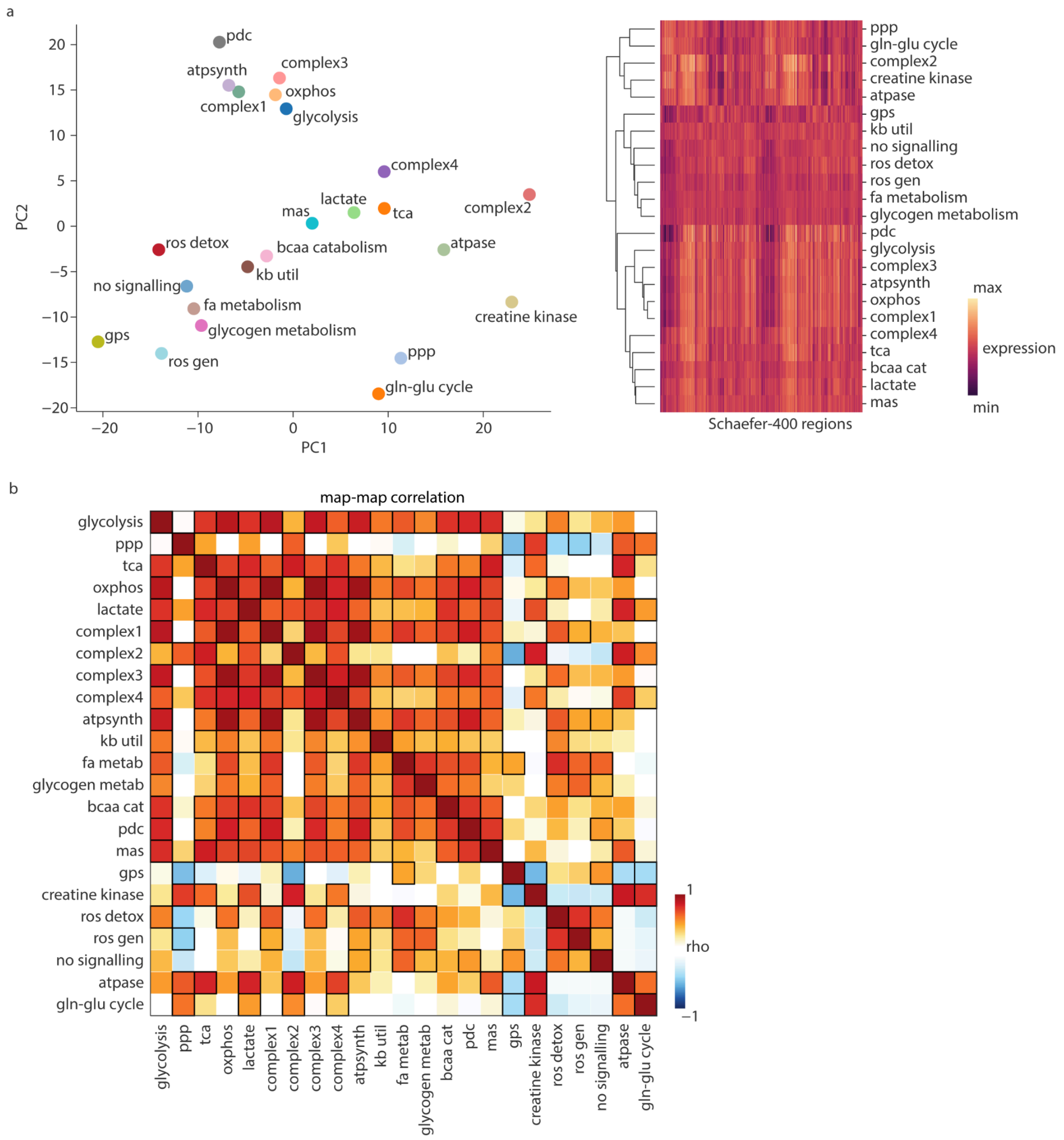


“Figure S17. Clustering analysis of energy maps. (a) Left: The clustering among the complete set of energy maps was carried out using PCA. Axes represent the first and second principal components. Right: hierarchical clustering of extended energy maps. Color bar represents expression values across the 400 cortical regions in the Schaefer parcellation. (b) Pair-wise correlation among the extended energy maps. Color bar represents Spearman’s correlation value. Bold edges indicate statistical significance tested against 10,000 spatial nulls and after FDR correction. atpsynth, ATP synthase complex; BCAA, branch-chained amino acids; pdc, pyruvate dehydrogenase complex; mas, malate-aspartate shuttle; gps: glycerol-3-phosphate shuttle; ros gen, generation of reactive oxygen species; no signaling, nitric oxide signaling; gln-glu cycle, glutamine-glutamate cycle”
